# Maturity-driven shifts in physicochemical, phytochemical and mineral composition of marula [*Sclerocarya birrea* (a. rich.) Hochst] fruit: a multivariate perspective on nutritional functionality

**DOI:** 10.3389/fnut.2026.1908448

**Published:** 2026-09-23

**Authors:** Mashudu Sikhavhakhavha, Nkosikhona Goodman Magwaza, Sabelo Shezi, Thanyani Ralulimi, Kuhlula Maluleke, Cynthia Kwena Marokane-Radebe, Makgafele Lucia Ntsoane, Mdungazi Knox Maluleke

**Affiliations:** 1 Leeto-La-Africa Community Engagement Scholarship Project (CU_1600), Department of Environmental Sciences, College of Agriculture and Environmental Sciences, University of South Africa, Tshwane, South Africa; 2 School of Health Systems and Public Health, Faculty of Health, University of Pretoria, Pretoria, South Africa; 3 Agricultural Research Council, Tropical and Subtropical Crops (ARC-TSC), Mbombela, South Africa

**Keywords:** underutilised fruit, maturity index, nutrition, postharvest quality, Zero-hunger

## Abstract

Approximately 50% of fruit is lost to postharvest spoilage due to the high metabolic rate of fresh produce. Reducing postharvest losses is crucial in eradicating hunger, reducing poverty and ensuring food security in line with Sustainable Development Goals 1 and 2. Understanding the optimal harvesting time for fruit is essential in maximizing its quality and extending shelf life. This study evaluated the effect of different maturity stages on the physicochemical, phytochemical and mineral profile of marula [*Sclerocarya birrea* (A. Rich.) Hochst] fruit. The fruit was harvested in the wild at Bushbuckridge-Lillydale (24.872°S 31.365°E) at different maturity stages (mature unripe, semi-ripe and fully ripe) during the summer season of 2025 and transported to the Eureka laboratory at Unisa for analysis. Upon arrival, the fruit were analyzed for physicochemical properties, including total soluble solids (TSS), titratable acidity (TA), pH, TSS/TA, phytochemical properties such as total phenolic content, flavonoids, carotenoids, vitamin C and mineral nutrients. The results revealed that green unripe fruit exhibited a superior total phenolic content of 14.5 GAE/100 g FW, followed by semi-ripe fruit at 10.6 GAE/100 g FW. The lowest total phenolic content was observed in fully ripe fruit at 6.9 GAE/100 g FW. A similar pattern was observed in total flavonoid content. In contrast, vitamin C was highest in fruit harvested at the semi-ripe stage. The findings from this study suggest that marula fruit exhibits varying phytochemical and nutritional contents at the different maturity stages. Therefore, it is essential to harvest it based on targeted phytochemicals or minerals.

## 1 Introduction

The Sustainable Development Goals (SDGs), an initiative of the United Nations, aim to eliminate poverty and hunger and to ensure good health and well-being for all people regardless of their economic background by 2030 (1, 2). The literature demonstrates that current crop production is sufficient to feed over 9 billion people globally (3). However, studies have shown that fruit postharvest losses constitute a significant global food concern and poses a substantial threat to food security, economic stability and environmental sustainability by destroying around 25 to 50% of the world’s fruit production prior to consumer access (4). A key factor contributing to these losses is the harvesting of fruit without following a ripeness indicators, resulting in postharvest losses (5, 6). Harvesting fruit prematurely typically results in underdevelopment, inferior flavor and increased vulnerability to shriveling, whereas harvesting it too late causes over-ripening and accelerated deterioration (7). The marula (*Sclerocarya birrea* (A. Rich.) Hochst) (Figure 1) fruit lacks a harvesting maturity index. This indigenous African fruit is reported to be rich in vitamin C and other minerals, such as calcium, potassium and phosphorus, all of which humans require daily to maintain their well-being (8–10). Rural and semi-rural cultures in Africa, especially in South Africa, typically consume the marula fruit as a snack and utilize it as a raw material for producing value-added products, including alcoholic drinks, jam, juice and nuts (9). This makes the marula a significant indigenous fruit that contributes to food and nutritional security among South African communities. Understanding and profiling its maturity index is crucial to minimizing losses during the harvest season. As a result, the study examined changes in the physicochemical and mineral composition of marula fruit at different ripeness stages (green, semi-ripe and fully ripe) to determine how ripeness served as a crucial indicator for horticultural harvesting and its potential benefits for human health and nutrition.

**Figure 1:**
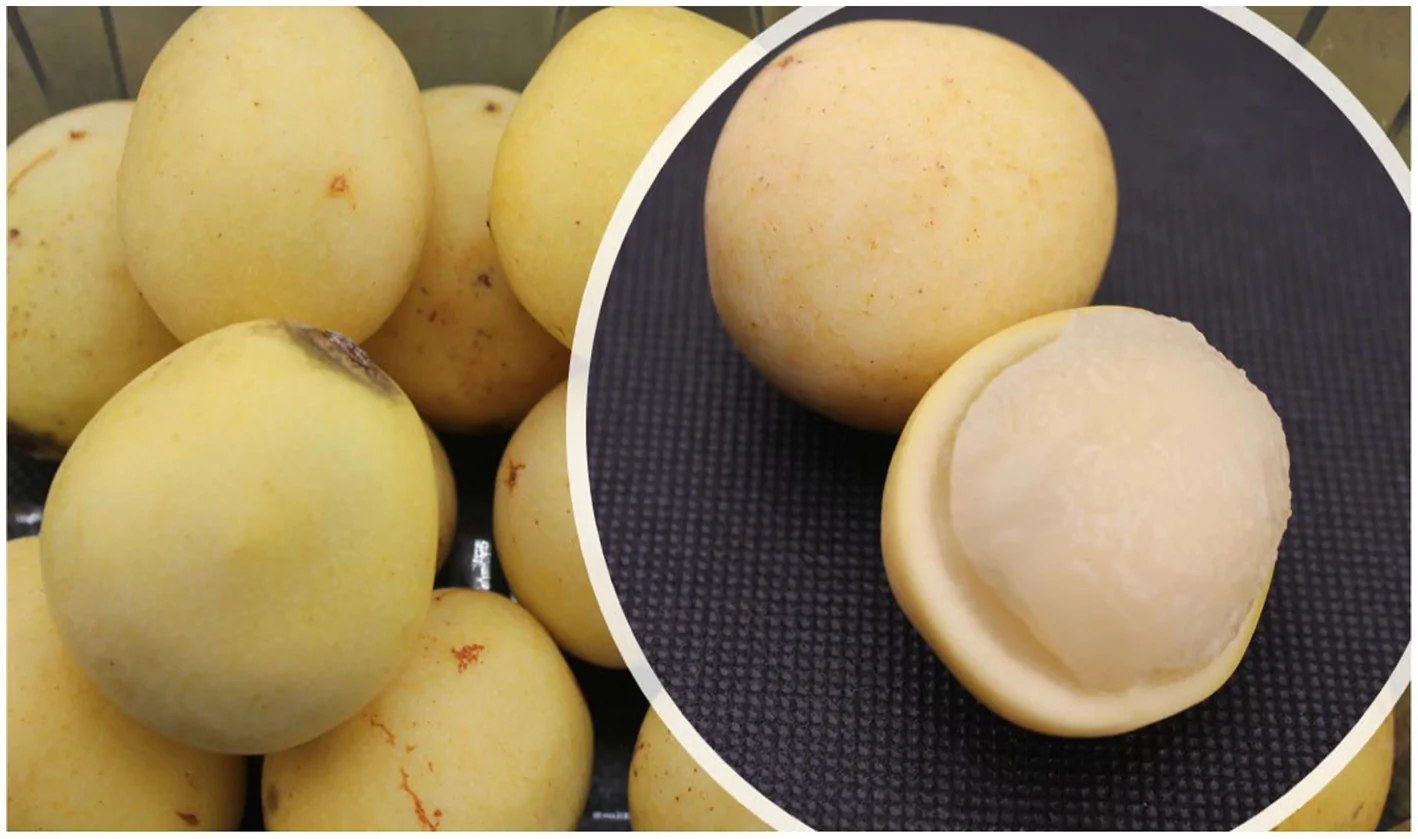
Fully ripe marula fruit.

## 2 Materials and methods

### 2.1 Fruit collection

Utilizing the methodology of (11), as adopted by (12), a slightly modified cluster randomized sampling strategy was employed to gather fruit in the wild at the Lillydale village in the Bushbuckridge area (24.872°S 31.365°E) during the summer season of 2025 as shown in Figure 2. The prevalence and utilization informed the selection of the study area. A total of 36 marula fruit trees were randomly selected for sampling in this investigation. Fruit harvested represented three stages of ripeness: (i) green unripe, semi-ripe, and fully ripe. Forty fruits were harvested from each tree, leading to an aggregate of 1,440 collected across different trees. To provide equitable representation in sampling, 12 trees were randomly selected and sampled for every maturity phase. The collected fruit were categorized based on their ripeness as determined by their external characteristics, including peel coloration, utilizing the color chart adopted by (9). Only fruit that displayed uniformity in color, size, shape, and free from physical damage or other evident flaws, were selected for sampling to assure consistency and decrease variability among samples.

**Figure 2:**
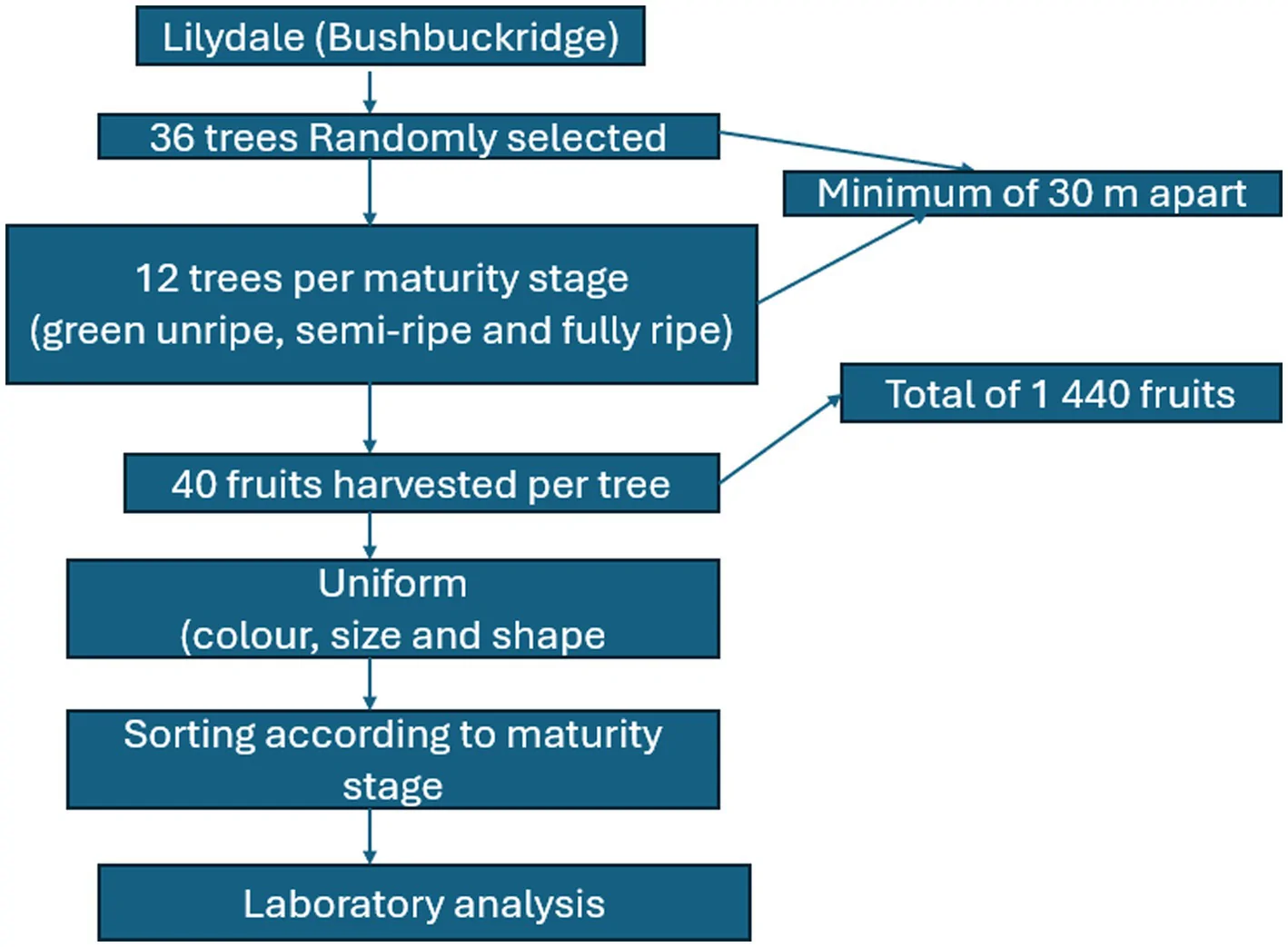
Illustrating of the sampling procedure that was adopted for marula fruit collection.

#### 2.1.1 Experimental design

The study used a completely randomized design (CRD), with fruit maturity stages (green unripe, semi-ripe and fully ripe) serving as the independent variable separated as presented in Figure 3. The dependent variables included (i) physicochemical properties such as (color, pH, titratable acidity, total soluble sugars, and sugar-to-acid ratio), (ii) phytochemicals including (beta carotene, total flavonoids, total phenols, and vitamin C), and (iii) minerals including (calcium, magnesium, phosphorus, sodium, copper, iron, and zinc). Fruit from each tree were used as experimental units in laboratory investigations, while sampled trees provided biological replications. This strategy allowed for comparisons between the three maturity stages (green unripe, semi-ripe and fully ripe), while reducing sample bias and assuring adequate replication for statistical analysis.

**Figure 3:**
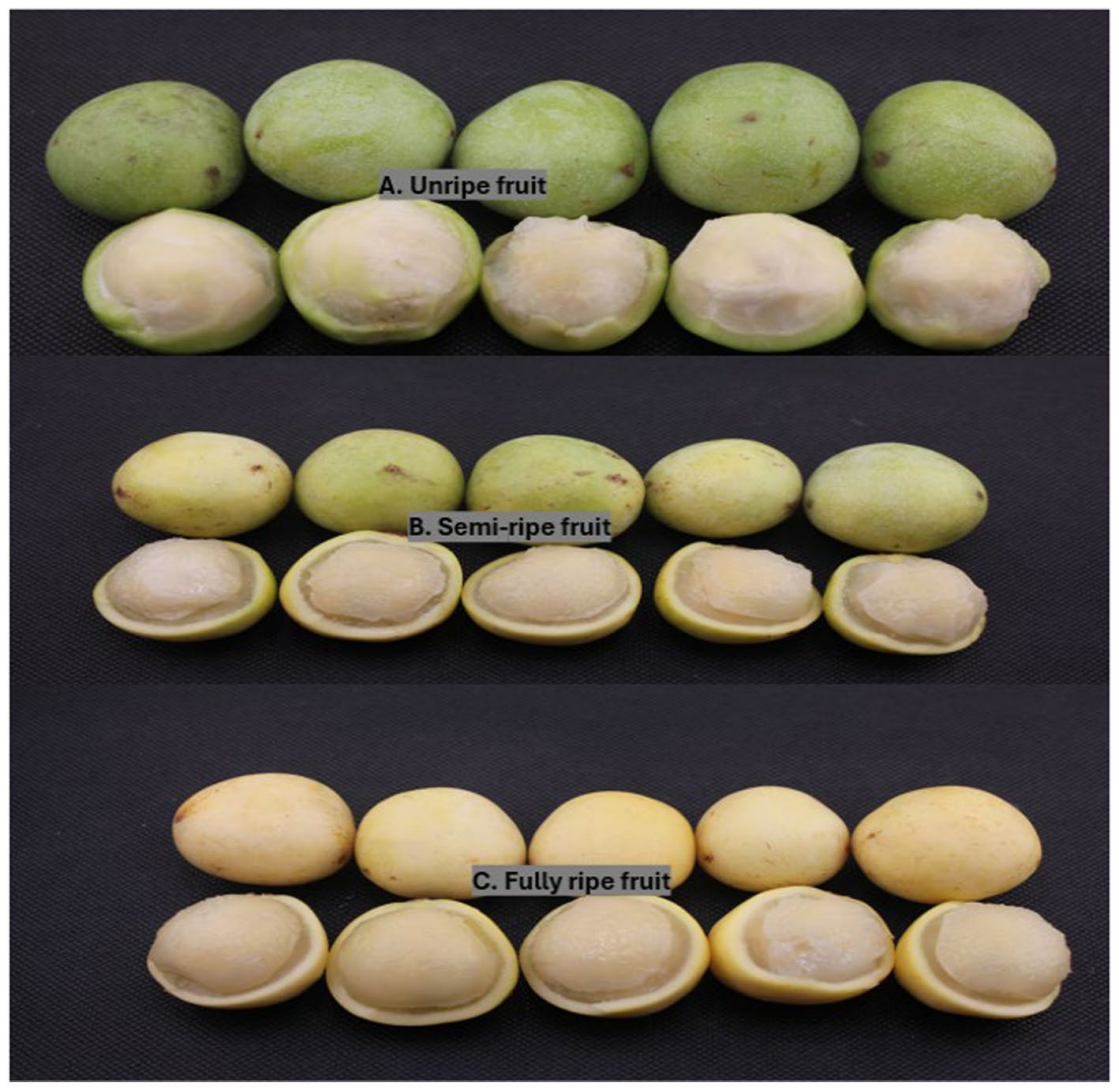
Marula fruit of different maturity stages. Unripe **(A)**. Semi-ripe **(B)**. Fully ripe **(C)**.

#### 2.1.2 Sample preparation

The harvested fruit were subsequently transferred to the Eureka laboratory facility at the University of South Africa, Florica Science campus (S 26° 9′2.52“, E 27° 54’ 9.504”). Upon arrival, fruit were hand sorted for uniformity in color, shape, size and flaws. Prior to sample preparation, the fruit were disinfected by rinsing with a 0.02% sodium hypochlorite (NaClO) solution for 3 min and air dried at room temperature. Following disinfection and air drying, the fruit was peeled using a sharp, sterilized knife to remove the pulp from the seeds. The experiment was conducted in strict accordance with the University of South Africa (Unisa), College of Agriculture and Environmental Sciences Research and Higher Degree Committee (Ethical number/Reference #: 2022/CAES HREC/104), as well as pertinent local and international regulations. It is worth to note that the results presented in this study are derived exclusively from laboratory analysis, as no clinical trial has been performed.

#### 2.1.3 Determining average recommended daily intake (ARDI)

To estimate the potential nutritional contribution of marula fruit, the concentration of each nutrient (expressed per 100 g fresh weight) was compared with published dietary values for reference. Average Recommended Daily Intake (ARDI) values were calculated from internationally recognized dietary reference recommendations for different population groups and were used only as a comparative reference to estimate the percentage contribution of a 100 g serving of marula fruit to daily nutrient requirements.

Average recommended daily intake (ARDI) formula:


ARDI=(Sumofallvalues)/(Number of values) (1)

The percentage contribution was calculated using Equation 2:


$$\text{Percentage contribution}\;(\%)=(\begin{array}{l} \text{Nutrient concentration}\;\\ \mathrm{per}\;100\;g÷\text{ARDI} \end{array})\times 100$$ (2)

The ARDI values presented in Table 1 are intended for comparative purposes only and should not be interpreted as recommended dietary allowances for any specific population group.

**Table 1:** Average recommended daily (mg) intake of phytochemicals and minerals.

Phytochemical/Mineral	ARDI	Reference
Beta carotene	4.5	(13)
Calcium	1,100	(10)
Copper	1.1	(14)
Iron	15.2	(15)
Magnesium	363	(16)
Phosphorus	369.8	(17)
Sodium	1,500	(18)
Total flavonoids	750	(11, 19)
Total phenols	959	(11, 20)
Vitamin C	92.5	(10, 20)
Zinc	9.1	(11, 21)

ARDI = Average recommended daily intake.

### 2.2 Determining color attributes

The fruit color attributes L, a*, b*, C and h were determined using the colorimeter (Lovibond® LC100, Japan) in CIELAB (L*, a* and b*) according to (22). The parameter L* presents the fruit’s lightness, a* is the hue range of colors red (+) and green (−) and b* is the hue range of colors yellow (+) and blue (−). The parameters C* and h represents the chroma and hue angle, respectively.

### 2.3 Determining chemical attributes

#### 2.3.1 Determining pH and titratable acid

Marula pulp was extracted from 18 fruits at each maturity stage, comprising three replicates of six fruits per replicate. The pH was determined using the pH meter (Prime-Flash Automatic Titrator, Steroglass Srl AS24, Italy) previously calibrated with buffer solutions (at pH 4 and 7). The TA was determined using the method of (23), in which 10 mL was added to 50 mL of distilled water and auto-titrated against 0.1 N NaOH solution to reach an endpoint pH of 8.2. The TA was expressed in g/L citric acid.

#### 2.3.2 Determining total soluble sugars

Total soluble solids were determined using a handheld digital refractometer (HI 96801 Refractometer, USA) and the results were expressed in °Brix according to (24). About two drops (~0.03 mL) of fruit juice were placed on the aperture of the refractometer, and the readings were recorded. The aperture was washed with distilled water and wiped with a paper towel after each reading.

#### 2.3.3 Determining sugar-to-acid ratio

The sugar-to-acid ratio was determined by dividing total soluble solids by titratable acidity, and the results were expressed as the ratio (25).


Total soluble sugars/Titratable acids=Sugar−to−acid ratio (3)

### 2.4 Determining phytochemical attributes

#### 2.4.1 Total flavonoids and phenolic content

Following the methodology established by (26) and (27), fresh marula fruit pulp samples at different maturity stages (green unripe, semi-ripe, fully ripe) were quantified utilizing the aluminum chloride colorimetric method. Fifty mg of marula fruit pulp were dissolved in 1 mL of methanol, mixed with 4 mL of distilled water, and subsequently combined with 0.3 mL of a 5% NaNO2 solution. After a 5-min incubation, 0.3 mL of 10% AlCl3 solution was added, and the mixture was allowed to stand for 6 min. The volume of the mixture was adjusted to 10 mL with double-distilled water after the addition of 2 mL of 1 mol/L NaOH solution. After 15 min of incubation with the combination, the absorbance was measured spectrophotometrically at 510 nm. Catechin served as the standard for the calibration curve, and total flavonoid content was expressed in milligrams of catechin equivalents per hundred grams of fresh weight (mg CE/100 g FW).

Regarding the total phenolic content, the method of (28), adopted by (29), was utilized with slight modifications. In summary, fresh pulp of marula fruit (green unripe, semi-ripe, fully ripe) was used to extract total phenol concentration, using gallic acid as a standard reference (Sigma, St. Louis, MO). An aliquot of the extract was oxidized using Folin–Ciocalteu reagent (2 N, Sigma, St. Louis, MO) at a 10:1 volume ratio. The samples were then incubated in 96-well microplates for 20 min at ambient temperature, and the absorbance was measured at 750 nm using a microplate reader (Synergy HT, Bio-Tek, Winooski, VT). The total phenolics were quantified as milligrams of gallic acid equivalents per gram of fresh weight (mg GAE/100 g FW).

#### 2.4.2 Beta carotene and vitamin C

For beta carotene, the method outlined by (27) and (24) adopted by was utilized with minor modification. In summary, a Prominence-in High Performance Liquid Chromatography (HPLC)-PDA model system with a sample chiller (LC-2030C, Shimadzu, Japan) was employed for the analysis. Analyses were performed in triplicate as follows: the extracted sample (0.1 g/mL) in ice-cold hexane: acetone (1:1, v/v) was vortexed for 2 min before centrifugation at 2000 rpm for 2 min. The resulting organic phase was decanted into a tube with saturated sodium chloride solution after being placed on ice. A colorless extract was obtained by re-extracting the remaining residue in a similar manner. Prior to injection into the HPLC, the organic phase of the extract was filtered through a 0.45 μm syringe filter. Chromatographic separation was achieved using a C18 Luna® column (150 4.6 mm, 5″) at 35 °C with a flow rate of 1 mL/min, an injection volume of 20 μL and a detection wavelength of 450 nm. The isocratic mobile phase comprised acetonitrile, dichloromethane and methanol (7:2:1). Peak identification and quantification of beta carotene were accomplished using calibration curves based on an authentic β-carotene standard (24). The amount of beta carotene was expressed as mg 100/g of fresh weight.

For vitamin C content, the method described by (26) was followed. Marula fruit pulp samples (0.5 g) were combined with 2.5 mL of the specified extractant solution. For MPA–acetic acid extraction, the solution comprised 3% MPA and 8% acetic acid, while for oxalic acid extraction, it contained 0.1% oxalic acid. Subsequently, the mixture was homogenized in a Politron PT 6000 high-speed blender (Kinematic AG, Switzerland) at 18000 g under ice and darkness for 1 min, followed by centrifugation at 9000 g (refrigerated at 4 °C) for 20 min. This entire process was repeated twice, and the resulting supernatants were combined. All extractions were conducted in triplicate, with measures taken to minimize light exposure and a temperature of 4 °C throughout the procedures. The vitamin content was quantified in milligrams per 100 grams of dry weight.

#### 2.4.3 Mineral constituents determination

For mineral analysis, expressed in milligrams per 100 grams fresh weight (mg/100 g FW), the procedure of (27), adopted by (29), was followed with minor modification (triplicates). In short, a dispersed microwave system (MLS 1200 Mega; Milestone Sr. l, Sorisole, Italy) was used to digest fresh pulp samples. Adjustments involved introducing 2 mL of HNO3 (67% analphur) and 1 mL of analytical-grade H202 into polytetrafluoroethylene vessels prior to measuring samples in triplicate (ranging from 15 to 25 mg) for each treatment. After digestion, each solution was diluted to 15 mL in a test tube containing deionised water, and ICP-MS was employed for result analysis. The ICP-MS apparatus (Agilent 7,700; Agilent Technologies, Tokyo, Japan) featured a quadrupole mass analyzer and an octapole reaction system (ORS 3). The calibration solution was prepared by appropriately diluting the single-element certified reference material (Analytika Ltd., Czech Republic; 1.000 0.002 g/L for each element) with deionized water (18.2 M.cm, Direct-Q; Millipore, France). The accuracy of the measurements was validated using certified reference material for water, TM-15.2, from the National Water Research Institution in Ontario, Canada. The analysis covered nutrient elements, such as calcium (Ca), magnesium (Mg), phosphorus (P), sodium, copper (Cu), iron (Fe) and zinc (Zn), and their contributions to average daily intake (Equation 1), were then determined as shown in Table 1 and Equation 2.

### 2.5 Statistical analysis

The data gathered in this research study was subjected to statistical analysis utilizing Genstat® analysis of variance (17th Edition, VSN International Ltd., Hertfordshire, United Kingdom). A Shapiro–Wilk test was employed to determine normality, while a Chi-square test was utilized to evaluate the homogeneity of the data. The Duncan’s Multiple Range Test (DMRT) was employed as a *post-hoc* analysis to differentiate the means after obtaining significant results in a one-way ANOVA at a 95% confidence interval.

### 2.6 Multivariate statistical analysis

Multivariate statistical analyses were performed using MetaboAnalyst 6.0 (Xia Lab, McGill University, Montreal, QC, Canada). Separate datasets comprising the physicochemical, phytochemical and mineral composition of marula fruit at different maturity stages were analyzed. Prior to analysis, the data were log transformed to improve normality and stabilize variance, followed by auto scaling (unit variance scaling) to ensure equal contribution of all variables regardless of their original measurement units. Principal component analysis (PCA) was performed as an unsupervised multivariate technique to reduce data dimensionality, visualize sample clustering, identify patterns associated with fruit maturity, and determine the variables contributing most strongly to sample discrimination. The percentage of variance explained by each principal component, together with the corresponding loading vectors, was used to interpret the relationships among variables and maturity stages. Hierarchical clustering analysis (HCA) was subsequently performed using Euclidean distance and Ward’s linkage method to complement the PCA by grouping samples with similar physicochemical characteristics. The HCA results were visualized as a heatmap to illustrate similarities among maturity stages and the measured variables.

## 3 Results and discussion

### 3.1 Effect of maturity stages on the chemical attributes of marula fruit

Table 2 presents the pH, TA, TSS and sugar-to-acid ratio of marula fruit harvested at different maturation stages (green unripe, semi-ripe and fully ripe). The study found a significant (*p* ≤ 0.001) difference among fruit harvested at different maturity stages. The observed trend revealed that the pH, TSS and sugar-to-acid ratio of marula fruit increased as the maturation stage progressed from green to fully ripe. For the pH, the results ranged from 3.1 to 4.6. Furthermore, in relation to other phases of fruit maturation, fully ripe fruit demonstrated a higher pH at 4.6, followed by semi-ripe fruit at 3.6. The lowest pH content of 3.1 was recorded in green unripe fruit.

**Table 2:** Physicochemical properties of marula fruit harvested at different maturity stages.

Maturity stage	pH	Total soluble sugars (°Brix)	Titratable acidity	Sugar-to-acid ratio (%)
Green unripe	3.1 ± 0.7^a^	5.9 ± 0.6^c^	35.8 ± 0.9^a^	0.16 ± 0.01^c^
Semi-ripe	3.6 ± 0^b^	10.6 ± 2.1^b^	27.1 ± 2.3^b^	0.46 ± 0.1^b^
Fully ripe	4.6 ± 0.2^c^	16.1 ± 0.8^a^	20.8 ± 1.9^c^	0.78 ± 0.2^a^
Grand mean	3.75	8	27.9	0.446
LSD0.05	0.405	1.588	2.899	0.074
*p*-value	0.001	0.001	0.001	0.001

Values present the mean ± SE, *n* = 6; different letters within a column indicate significant differences (*p* ≤ 0.05).

It has been established that green or unripe fruit accumulates more acidity at the early stages of development, mainly as a defensive mechanism against abiotic and biotic stress (30). The high acidity at this level is associated with the high levels of organic acids, which are later broken down into simpler sugars during fruit maturity and ripening (84). In this study, the variation between the highest pH (4.6) and the lowest (3.1) was 1.5, a significant difference. The fact that plants acquire more organic acids throughout cell development for defense and gradually accumulate sugar molecules as they mature could have been the potential attribute to variation in acidity (low pH content) in the early stage of marula fruit (green unripe) as opposed to the semi-ripe to full maturity stages. These findings are consistent with those of (31), who reported significant differences in the acidity of tomato fruits harvested at different maturation stages.

The observed trend showed that the titratable acidity of marula fruit decreased as the maturation stage progressed from green to fully ripe. The results showed that the TA ranged from 20.8 to 35.8. Furthermore, in relation to other phases of fruit maturation, green unripe fruit demonstrated a higher TA at 35.8, followed by semi-ripe fruit at 27.1. The lowest TA of 20.8 was recorded in fully ripe fruit.

It has been reported that green or unripe fruit accumulates more organic acids, as they are predominantly produced early in the developmental stage of maturity as a defensive mechanism against abiotic and biotic stress (32). Some of these compounds contribute positively towards strengthening the fruit’s morphological structures associated with protection during growth, with their concentrations decreasing as the fruit softens due to increased sugar accumulation as it matures or ripens (30). In this investigation, the difference between the highest TA content (35.8) and the lowest (20.8) is 15 g/L, a significant margin. The fact that plants acquire more organic acids for defensive purposes during cell development, along with the gradual buildup of sugar molecules as the fruit matures, could have been the possible attribute to the heightened titratable acidity found in the initial stage of marula fruit (green unripe) relative to its semi-ripe and fully mature stages. These findings are in line with those of (31), who also reported a significant variation in the TA of strawberry, sweet cherry and mulberry fruit harvested at different maturation stages.

The TA and pH are quantitative measures of acidity in fruit. The TA is quantified through titration and expressed as the percentage of the predominant organic acids, such as citric, malic, lactic, tartaric or acetic acid, depending on the fruit for which the acidity is quantified (33). For example, TA is expressed as citric acid in citrus fruit, whereas in apples, it is expressed as malic acid. TA strongly correlates with the number and concentration of organic acids; as a result, it increases in green mature fruit and decreases as it gets used up during fruit ripening. TA directly influences the flavor of fruit, as higher concentrations of organic acids alter its flavor and, consequently, its organoleptic quality (33). Therefore, in a fresh produce market, the TA determines the flavor, an important quality component.

For total soluble solids, the results ranged from 5.9 to 16.1%. Furthermore, in relation to other phases of fruit maturation, fully ripe fruit demonstrated a higher TSS content at 16.1%, followed by semi-ripe fruit at 10.6%. The lowest total soluble solids of 5.9% was recorded in green unripe fruit. It has been established that green or unripe fruit accumulates less soluble sugars and more acids, tannins and alkaloids as they are predominantly produced early in the developmental stage of maturity as a defensive mechanism against abiotic and biotic stress (30). The TSS is the most important flavor component in most fruits as it correlates with sweetness (87). In this investigation, the difference between the highest TSS content (16.1) and the lowest (5.9) is 10.2%, a significant margin. The fact that plants acquire more acidity throughout cell development for defense and gradually accumulate sugar molecules as they mature appears to be a possible attribute of lower TSS content in the early stage of marula fruit (green unripe) as opposed to the semi-ripe to full maturity stages. These findings are consistent with those of (31), who reported a significant variation in the TSS of strawberry, sweet cherry and mulberry fruit harvested at different maturation stages. The TSS normally increases with maturity and ripening in most fruit, mainly because starch, organic acids and other compounds are broken down for the synthesis of simpler sugars such as sucrose and fructose during fruit ripening (5, 31). Furthermore, the fruit juice continuously gets concentrated during postharvest storage because of the loss of moisture from the fruit through transpiration (34).

For the sugar-to-acid ratio, the results ranged from 0.16 to 0.78. Furthermore, in relation to other phases of fruit maturation, fully ripe fruit demonstrated a higher sugar-to-acid ratio at 0.78, followed by semi-ripe fruit at 0.46. The lowest sugar-to-acid ratio of 0.16 was recorded in green unripe fruit. It has been established that green or unripe fruit accumulates more acids and secondary metabolites as they are predominantly produced early in the developmental stage of maturity as a defensive mechanism against abiotic and biotic stress (30). In this investigation, the difference between the highest sugar-to-acid ratio (0.78) and the lowest (0.16) is 0.62, a significant margin. The fact that plants acquire a greater proportion of acids throughout cell development for defense and gradually accumulate sugar molecules as they mature appears to be a possible attribute of the lower sugar-to-acid ratio in the early stage of marula fruit (green unripe) as opposed to the semi-ripe to full maturity stages. These findings are consistent with those of (35), who found variations in the sugar-to-acid ratio of strawberry fruit harvested at different maturation stages. In this study, the TSS/acid ratio increased as strawberries ripened.

Regarding the importance of the pH, TA, TSS and TSS/TA ratio in the quality of fruit and on human health, these physicochemical properties control the overall sugar and acid levels in fruits. The TSS represents all the water-soluble substances in fruit juice or extracts, primarily sugars (glucose, fructose, and sucrose), nutrients, organic acids (citric and malic), soluble amino acids, minerals, vitamins and other small soluble compounds (36). Soluble sugars contribute about 85% of the TSS, which is why the TSS is strongly correlated with sweetness 38 (37, 38). Soluble sugars are a substantial source of energy/calories, an essential nutritional requirement for the human body (39, 40) further reported that the major function of sugars is to provide energy and sweetness in food products. It further serves an important role in preserving the color and firmness of fresh produce. On the other hand, titratable acidity reflects the dominant organic acids in fresh produce. A change in the quantity and proportion of organic acids affects the flavor of fruit; therefore, the TA directly alters the flavor, and hence the organoleptic quality of fruit (41). The composition and proportions of sugars and acids determine the flavor and hence the organoleptic quality of fruit (42).

To further explain the relationships among physicochemical parameters and their collective influence across maturity stages, principal component analysis (PCA) and hierarchical clustering were performed (Figures 4, 5). The PCA biplot showed a clear separation of samples according to maturity stage, with the first two principal components (PC1 and PC2) explaining 95.7 and 3.7% of the total variance, respectively (Figure 4). PC1 represented the primary maturity gradient, separating green unripe fruit from fully ripe fruit based on their physicochemical characteristics. The corresponding PCA loading plot (Supplementary Figure S1) showed that titratable acidity (TA) contributed positively to PC1, whereas total soluble solids (TSS) and the sugar-to-acid ratio contributed negatively, indicating that these variables were the principal drivers of sample discrimination. This distribution reflects the metabolic changes associated with fruit ripening, during which organic acids are progressively metabolized while soluble sugars accumulate through carbohydrate metabolism, resulting in improved sweetness and a higher sugar-to-acid ratio. Similar physicochemical transitions during fruit maturation have been reported in marula and other climacteric fruits, where sugar accumulation and acid degradation are key determinants of fruit quality and palatability (43). The heatmap in Figure 5, further confirmed this clustering pattern, showing an opposite relationship between acidity and sugar-related parameters across ripening. These findings indicate a coordinated biochemical transition from acid-dominated to sugar-dominated profiles during fruit maturation, which is expected to enhance fruit flavor and consumer acceptability.

**Figure 4:**
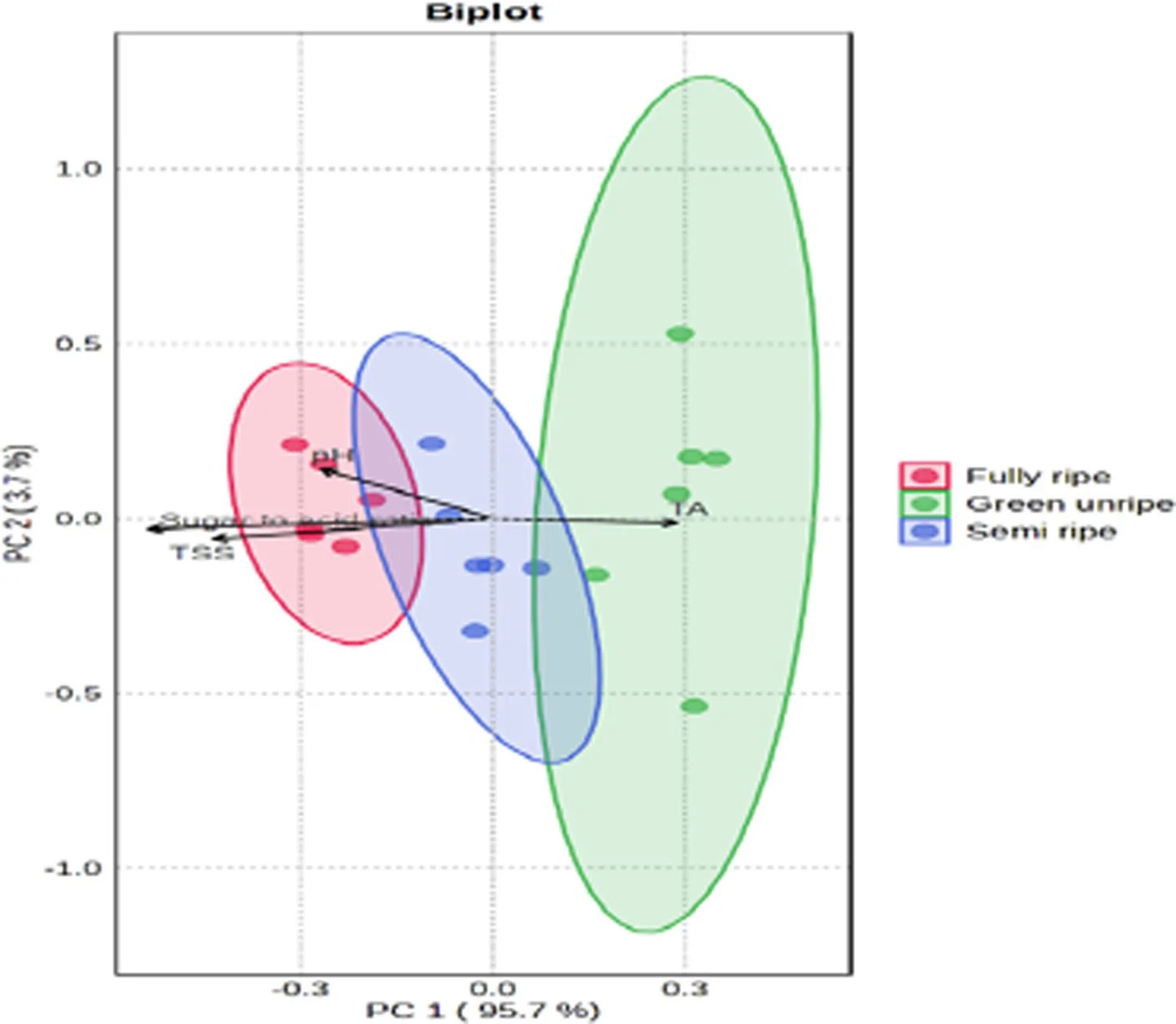
Principal component analysis (PCA) biplot showing the distribution of physicochemical parameters (pH, TSS, TA and sugar-to-acid ratio) across different maturity stages of marula fruit. The plot illustrates the separation of samples and the contribution of variables to the observed variation.

**Figure 5:**
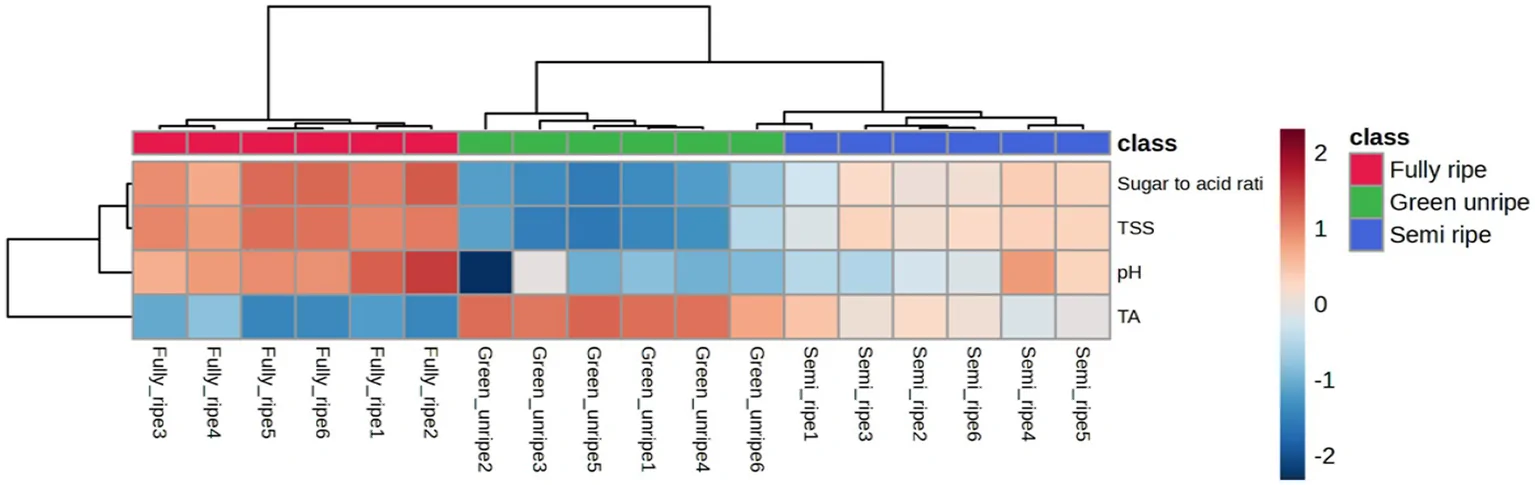
Heatmap representing the relative variation in physicochemical attributes of marula fruit across maturity stages. Color intensity indicates the magnitude of each parameter, with hierarchical clustering showing grouping patterns among samples and variables.

### 3.2 Effect of maturity stages on the phytochemical attributes of marula fruit

#### 3.2.1 Total flavonoids and phenols

Figure 6 depicts the total flavonoids and phenolic content of marula fruit harvested at various maturation stages (green unripe, semi-ripe and fully ripe). The study found a significant (*p ≤* 0.001) difference among fruit harvested at different maturity stages. The observed trend, further supported by multivariate analysis, revealed that the total flavonoid and phenol content of marula fruit reduced as the maturation stage progressed from green to fully ripe. For the total flavonoid content, as depicted in Figure 6A, results showed that it ranged from 1.8 to 11.7 mg CE/100 g FW. Furthermore, relative to other stages of fruit maturation, green unripe fruit demonstrated a higher flavonoid content at 11.7 CE/100 g FW, followed by semi-ripe fruit at 5.8 mg CE/100 g FW. The lowest total flavonoid content of 1.7 mg CE/100 g FW was observed in fully ripe fruit.

**Figure 6:**
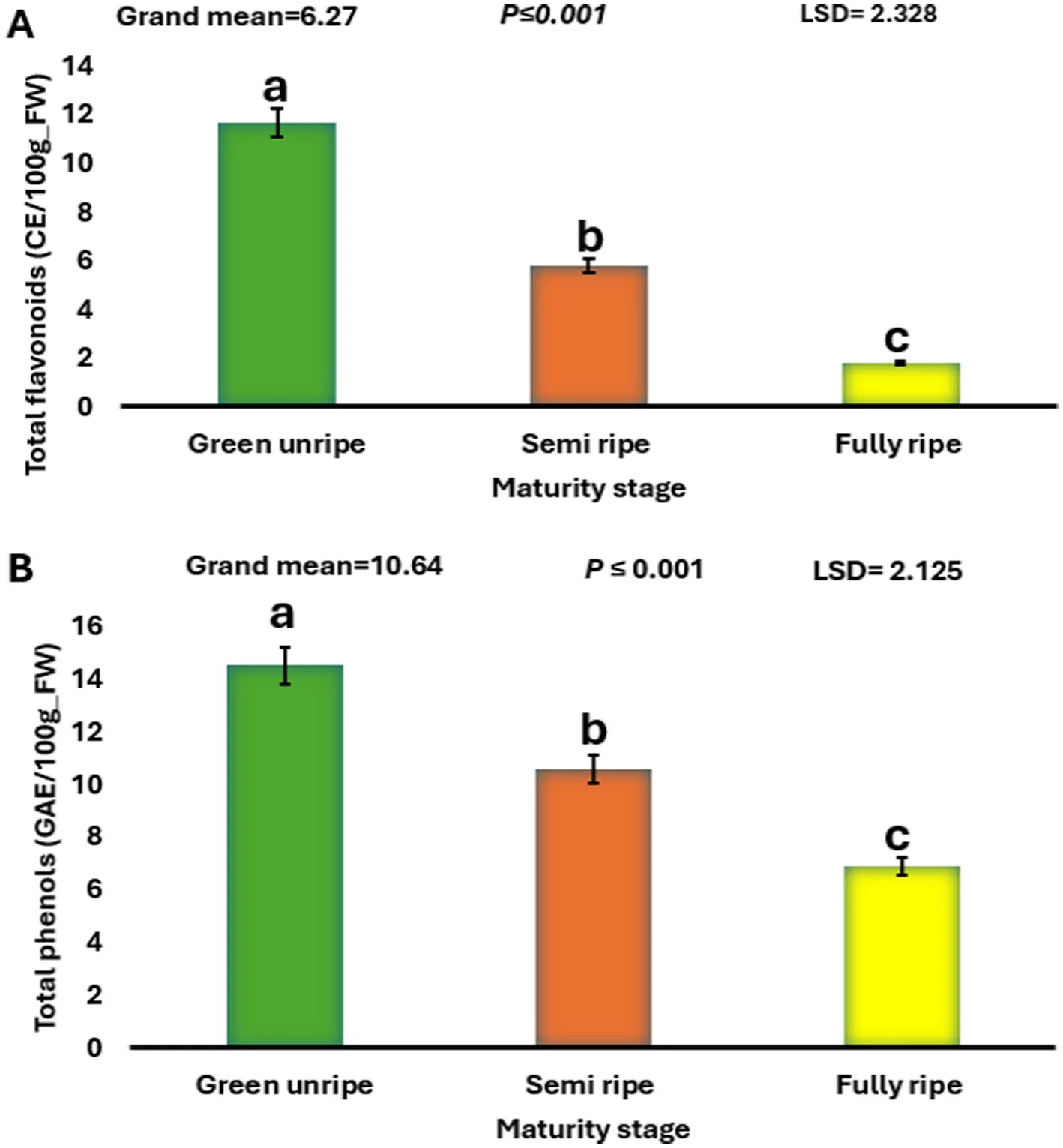
Total flavonoids **(A)** and phenols **(B)** of marula fruit harvested at different maturity stages. Values present the mean ± SE, *n* = 6, different letters indicate significant difference at *p* ≤ 0.05.

It has long been established that green or unripe fruit accumulates more flavonoids as they are predominantly formulated early in the developmental stage of maturity as a defensive mechanism against abiotic and biotic factors, including pests, diseases and UV damage (30). These compounds also act as structural components; hardening cell walls and their concentrations decrease as fruit softens due to increased sugar accumulation as maturity or ripening increases (32). In this investigation, the difference between the highest (11.7) and lowest flavonoid content (1.7) is 10 mg CE/100 g FW, a significant margin. The fact that plants acquire more flavonoids throughout cell development for defense and gradually accumulate sugar molecules as they mature appears to be a direct cause of higher flavonoid content in the early stage of marula fruit, as opposed to the semi-ripe to full maturity stage. These findings are consistent with those of (43), who reported variations in flavonoid concentration in plum fruit harvested at different maturity stages.

The results for total phenols in Figure 6B demonstrate a range of 6.9 to 14.5 mg GAE/100 g FW. Moreover, comparative analysis indicates that green unripe fruit exhibited a superior total phenolic content of 14.5 mg GAE/100 g FW, followed by semi-ripe fruit at 10.6 GAE/100 g FW. The lowest total phenolic content was observed in fully ripe fruit at 6.9 mg GAE/100 g FW. Total phenolic content (TPC) is predominantly accumulated in green unripe fruit as a defensive mechanism against biotic and abiotic stressors, such as UV radiation and cold, via the phenylpropanoid pathway (11). The buildup is facilitated by the rate-limiting enzyme phenylalanine ammonia-lyase (PAL), which transforms primary phenylalanine into secondary phenolic metabolites (44). In this investigation, the ability of plants to protect young to semi-mature fruit during structural and cellular development against abiotic factors, including adverse environmental stresses such as heat, cold and UV light, could have directly contributed to superior total flavonoid levels in green unripe fruit compared to those in semi-ripe to fully ripe fruit. These findings correlate with those of (45), who reported varying total phenol levels in peach fruit harvested at differing maturity stages.

In human health and nutrition, total flavonoids and phenols are essential plant-derived phytochemicals that provide robust antioxidant and anti-inflammatory properties. These compounds play a pivotal role in protecting body cells from radiation-induced damage and in preventing various chronic diseases (20, 46). In this investigation, the variation between the highest total flavonoid content (11.7) and the average recommended daily intake (750) is 738.3 mg, while the variation between the highest total phenols (14.5) and the average recommended daily intake of total phenols (959) is 944.5 mg. This could imply that consuming marula fruit pulp derived from green unripe fruit could contribute around 1.6% of the total flavonoids required by humans daily, while the fruit pulp total phenolic content would contribute approximately 1.5%. Although the values obtained from this investigation are lower, consumption of green, unripe marula fruit could potentially help curb conditions such as frequent colds, constant bruising and poor wound healing, which are associated with low levels of flavonoids and phenols in the human diet (47).

Multivariate analysis of phytochemical attributes (Figures 7, 8) revealed distinct clustering patterns corresponding to fruit maturity stages. The PCA biplot revealed a clear separation of samples by maturity stage, with the first two principal components (PC1 and PC2) accounting for 90.5 and 6.6% of the total variance, respectively (Figure 7). Vitamin C was more closely associated with the green-to-semi-ripe stages, whereas beta carotene showed stronger alignment with the fully ripe stage. The association of vitamin C with early developmental stages reflects its role in protecting fruit tissues against oxidative stress and supporting active cellular metabolism (48). However, its concentration may decline during advanced ripening due to oxidative degradation and reduced biosynthetic activity (48). In contrast, the alignment of beta carotene with later maturity stages indicates enhanced accumulation during ripening, contributing to overall improved nutritional and fruit quality attributes (24). The heatmap (Figure 8) further illustrated a progressive decline in phenolic compounds alongside an increase in beta carotene during ripening, while vitamin C remained relatively higher in the early to mid-stages. Collectively, these findings suggest a metabolic shift from defense-related secondary metabolite accumulation in early stages to nutritional enhancement and fruit quality improvement in later stages.

**Figure 7:**
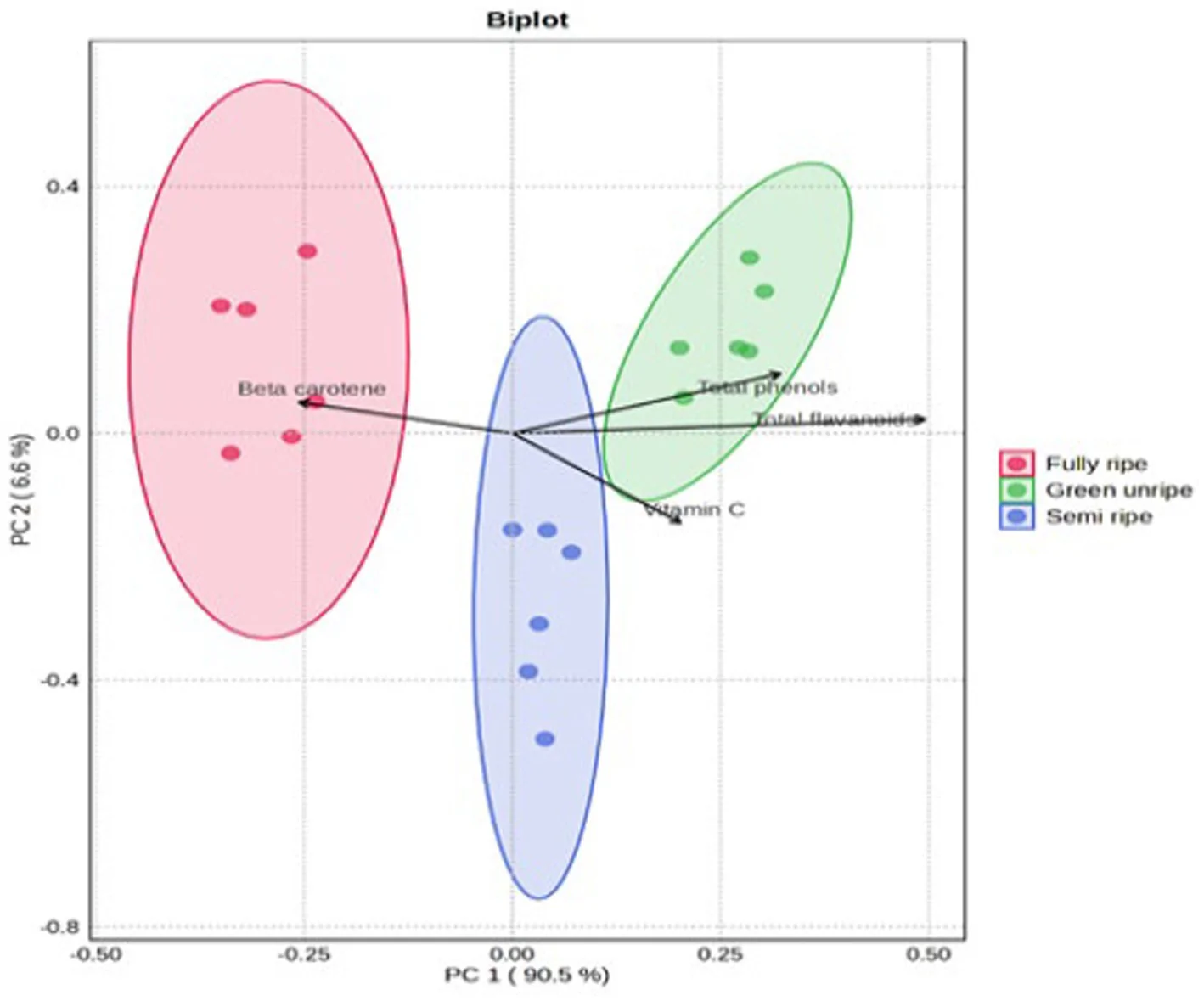
PCA biplot illustrating the variation in phytochemical composition (total phenols, flavonoids, beta-carotene, and vitamin C) across marula fruit harvested at different maturity stages.

**Figure 8:**
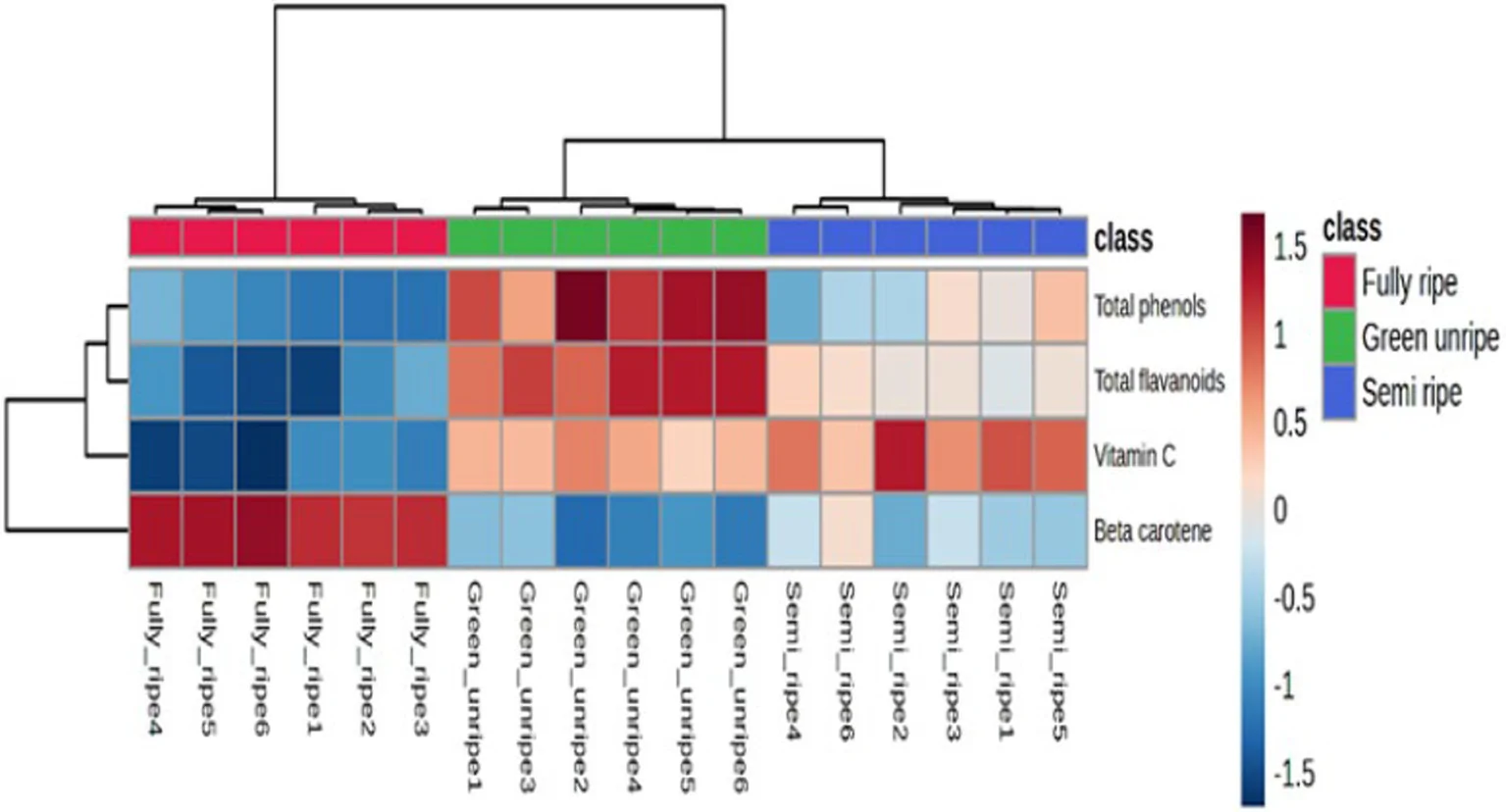
Heatmap showing the distribution and clustering of phytochemical compounds across different maturity stages of marula fruit.

#### 3.2.2 Beta carotene and vitamin C

Figure 9 depicts the beta carotene and vitamin C content of marula fruits harvested at different maturity stages (green unripe, semi-ripe and fully ripe). The study results illustrated a significant (*p ≤* 0.001) variation among fruit harvested at different maturity stages. For beta carotene Figure 9A, results indicated a range of 51.7 to 109 mg/100 g FW. Furthermore, the results demonstrated that fruit harvested at the green unripe stage had the lowest beta carotene concentration of 51.7 mg/100 g FW, followed by semi-ripe fruit at 87.1 mg/100 g FW. The maximum beta carotene content of 109 CE mg/100 g FW was observed in fully ripe fruit compared to other stages. It has been well established that the concentration of beta carotene in fruit normally peaks during the advanced phases of maturation and ripening (49). Fruit goes from the early embryonic stages to full ripeness, during which chloroplasts convert into chromoplasts, leading to a significant rise in orange and yellow coloring (50). In this investigation, analysis revealed a difference of 57.3 mg/100 g FW between the highest beta carotene content (109 mg in fully ripe fruit) and the lowest (51.7 mg in green unripe fruit), indicating a significant variation. As a result, the findings of this study suggest that the beta carotene content in marula fruit increases with advancing maturation stages, attributed to biochemical transformations that convert chloroplasts into chromoplasts, resulting in a color transition from green (unripe) to yellow (fully ripe). Similar findings were observed by (51), who identified maturity phases as a significant factor influencing differences in the beta carotene concentration of the fruits.

**Figure 9:**
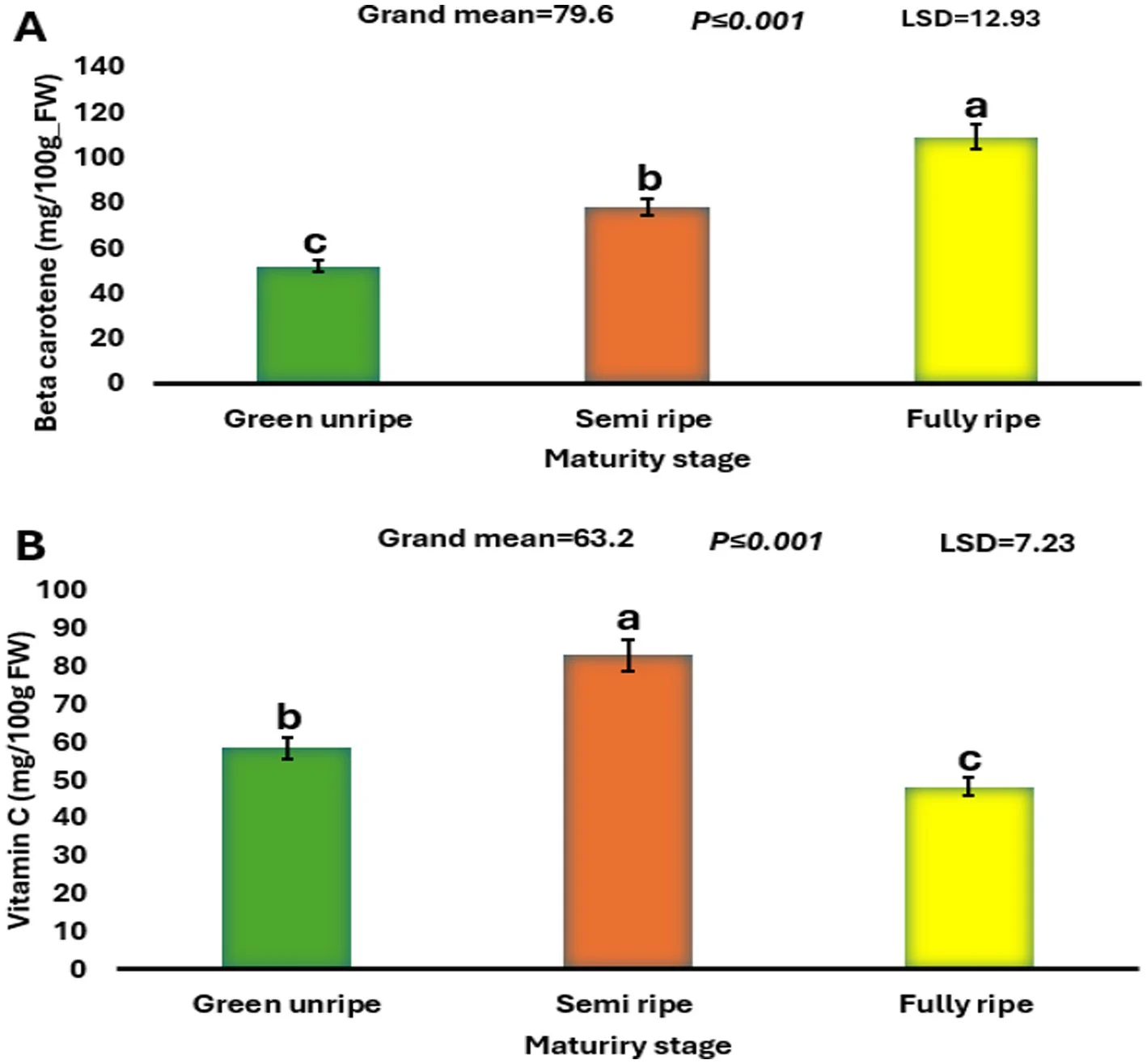
Beta carotene **(A),** and vitamin C **(B)** of marula fruit harvested at different maturity stages. Values present the mean ± SE, *n* = 6, different letters indicate significant difference at *p* ≤ 0.05.

In terms of human health and nutrition, beta carotene, predominantly derived from fruit, is a crucial dietary source of vitamin A, vital for maintaining optimal vision, bolstering immune function and regulating cellular growth (20). This study revealed a difference of 104.5 mg between the highest beta carotene content (109 mg) and the average recommended daily consumption (4.5 mg). Furthermore, this suggests that marula pulp from fully ripe fruit could provide above 100% of beta carotene required by humans daily. The relatively high beta carotene concentration observed in fully ripe marula fruit suggests that it may potentially serve as an important dietary source of provitamin A. However, the contribution of marula fruit to human health depends on nutrient bioavailability, dietary intake, and individual nutritional status, which were not evaluated in the present study.

Regarding vitamin C Figure 9B, results indicated that vitamin C content ranged from 58.4 to 83.1 mg/100 g FW. Furthermore, results demonstrated that fully ripe fruit had the lowest vitamin C content at 48.2 mg/100 g FW, followed by green unripe fruit with 84.4 mg/100 g FW. Compared with other maturity stages, semi-ripe fruit exhibited a higher content at 83.1 mg/100 g FW. The accumulation of phytochemicals, such as vitamin C, is directly influenced by different phases of fruit development. For example, in certain fruit, the concentration often increases from the green to semi-ripe stages to facilitate early cell division and cell wall construction (48). However, as the fruit ripens, softening typically occurs, leading to a steady decline in total content due to the oxidation process (52). In this investigation, the analysis revealed a disparity of 34.9 mg/100 g FW between the highest vitamin C content (83.1 mg in semi-ripe fruit) and the lowest (48.2 mg in fully ripe fruit). Values obtained from this investigation indicate that the vitamin C content of marula fruit increases during the intermediate stage to support both cell wall construction and division but subsequently decreases significantly as the fruit softens due to oxidation associated with ripening. These findings align with those of (53), who reported alterations in the phytochemical composition of grapes fruit with different maturity stages.

In terms of human health and nutrition, vitamin C (ascorbic acid) is a vital, water-soluble nutrient that humans cannot synthesize (11). It is crucial for tissue development and immune function and serves as a potent antioxidant that safeguards cells from damage (85, 86). In this investigation, the analysis reveals a discrepancy of 9.2 mg between the maximum vitamin C content (83.1 mg) and the average recommended daily intake (92.5 mg), indicating that pulps from semi-ripe marula fruit would potentially contribute about 90% of vitamin C required by humans daily. Furthermore, the findings of this study suggest that the consumption of semi-ripe marula fruit may potentially assist to prevent conditions such as chronic fatigue, bleeding gums, bruising and impaired wound healing, which are linked to insufficient vitamin C in the human diet (54, 55).

#### 3.2.3 Multivariate analysis of phytochemical changes during marula fruit maturation

Multivariate analysis of phytochemical attributes (Figures 7, 8) revealed distinct clustering patterns corresponding to fruit maturity stages. The PCA biplot showed a clear separation of samples according to maturity stage, with the first two principal components (PC1 and PC2) explaining 90.5 and 5.6% of the total variance, respectively (Figure 7). The corresponding PCA loading plot (Supplementary Figure S2) indicated that total flavonoids and total phenols were the principal contributors to the positive direction of PC1, whereas β-carotene loaded in the opposite direction, reflecting contrasting phytochemical accumulation patterns during fruit ripening. Vitamin C exhibited the strongest loading on PC2, demonstrating that it was the primary variable contributing to variation along the second principal component. The PCA biplot further showed that vitamin C was more closely associated with the green-to-semi-ripe stages, reflecting its important role in protecting developing fruit tissues against oxidative stress and supporting active cellular metabolism during early fruit development (48). Similarly, the greater contribution of total phenols and flavonoids to PC1 is consistent with their recognized roles as antioxidant metabolites involved in plant defense and protection against environmental stress, with concentrations generally declining as ripening progresses due to shifts in secondary metabolism. In contrast, β-carotene showed a stronger association with the fully ripe stage, indicating enhanced carotenoid accumulation during ripening, which contributes to fruit pigmentation, antioxidant capacity and improved nutritional quality (24). The heatmap (Figure 8) further supported these observations by showing a progressive decline in phenolic compounds alongside an increase in β-carotene during fruit maturation, while vitamin C remained relatively higher during the early to intermediate stages. Collectively, these findings demonstrate a coordinated metabolic transition from defense-related antioxidant compounds during early fruit development to carotenoid accumulation during ripening, reflecting the physiological and biochemical changes associated with fruit maturation and the enhancement of fruit quality.

### 3.3 Effect of maturity stages on the mineral attributes of marula fruit

#### 3.3.1 Macro-minerals composition

Table 3 presents the levels of calcium, magnesium, phosphorus and sodium in marula fruit at different stages of ripeness (green unripe, semi-ripe and fully ripe). The analysis indicated a significant (*p* ≤ 0.001) difference in macro-mineral (calcium, magnesium, phosphorus, sodium) content across maturity stages. For calcium, results ranged from 55.7 to 104.5 mg/100 g FW. Additionally, results depicted that fully ripe fruit had the lowest calcium content at 55.7 mg/100 g FW, followed by semi-ripe fruit at 73.2 mg/100 g FW. Compared with other stages, green unripe fruit at 104.4 mg/100 g FW had the highest content.

**Table 3:** Effect of different maturity stages on the macro-mineral content of marula fruit.

**Maturity stage**	**Calcium**	**Magnesium**	**Phosphorus**	**Sodium**
Green unripe	104.5 ± 10.3^a^	61.3 ± 2.3^c^	207.6 ± 9.3^a^	22.4 ± 2.6^a^
Semi-ripe	73.2 ± 15.6^b^	88.7 ± 1.6^b^	90.7 ± 2.6^b^	15.2 ± 0.9^b^
Fully ripe	55.7 ± 7.8^c^	105.1 ± 6.9^a^	51.8 ± 1.8^c^	10.1 ± 1^c^
Grand mean	77.8	85	116.7	15.85
LSD0.05	9.33	8.44	37.64	2.357
*P*-value	0.001	0.001	0.001	0.001

Values present the mean ± SE, *n* = 6; different letters within a column indicate significant differences (*p* ≤ 0.05).

It has been well established that calcium abundance in fruit is normally acquired during the early stages, as it assists in auxin production for seed development, stabilizes cell membranes and facilitates the formation of structural components (56). For this investigation, the difference between the highest calcium content (104.5) in green unripe fruit and the lowest (55.7) was 48.8 mg/100 g FW, which varied by a reasonable margin. Therefore, the higher calcium content in green unripe fruit could be attributed to the fact that there is a high demand for calcium as a mineral responsible for cell membrane stabilization and structural formation (57) but decreases as the fruit expands through a dilution effect caused by water uptake and sugar formulation. These findings are consistent with those of (58), who observed variation in calcium content across macadamia nut fruit at different development stages.

Regarding human health and nutrition, calcium is recognized as the most abundant mineral in the human body, primarily concentrated in bones and teeth, where it provides essential structure and strength (59). Beyond supporting skeletal integrity, calcium is also vital for several key metabolic functions (60). In this investigation, the variation between the highest calcium content observed (104.5 mg) and the average recommended daily intake (1,100 mg) is 995.5 mg. This implies that pulp sourced from green unripe marula fruit would contribute around 9.5% of calcium required by humans daily. Even though the calcium concentrations recorded in this study fall short of the average recommended daily intake, the consumption of green unripe marula fruit could potentially mitigate ailments such as muscle spasms, bone weakness, and abnormal heart rhythms, which are linked to low calcium intake in human diet (61).

Regarding magnesium content, results ranged from 61.3 to 105.1 GAE/100 g FW. Green unripe fruit at 61.2 mg/100 g FW had the lowest magnesium content, followed by semi-ripe fruit at 88.7 mg/100 g FW. Superior magnesium content was observed in fully ripe fruit harvested at 105 mg/100 g FW. It has long been established that magnesium is critical for fruit development, serving as a core component of chlorophyll, which is necessary for photosynthesis and as a key activator of enzymes responsible for carbohydrate metabolism and nutrient movement (62). For this study, the variation between the highest magnesium content (105) at the fully ripe stage and the lowest (64.3) was 40.7 mg/100 g FW, which was significant. Therefore, due to its mobility, an increase in magnesium content in fully ripe fruit could be attributed to the fact that, as the fruit matures, complex carbohydrates break down into simple sugars, causing fluctuations in cellular moisture as resources are allocated. These findings concur with those of (63), which demonstrate that magnesium plays a significant role in enhancing sweetness, color development and overall ripening as fruit maturity advances.

In terms of human health and nutrition, magnesium is a vital mineral and electrolyte that powers a variety of metabolic reactions in the human body (20). It is also essential for energy production, muscle and neuron function, blood sugar regulation and DNA synthesis (64). In this study, the difference between the highest magnesium content (105.1 mg) in fully ripe fruit and the average recommended daily intake (363 mg) was 257.9 mg. This indicates that pulps derived from completely fully ripe marula fruit could potentially provide around 29% of the daily magnesium requirement by humans. Despite the magnesium concentrations being beneath the ARDI, the consumption of fully ripe marula fruit could perhaps potentially assist to mitigate conditions like muscle cramps, constant fatigue, weakness, and diminished appetite, which are associated with insufficient magnesium intake in the human diet (65).

In terms of phosphorus content, the study found values ranging from 51.8 to 207.6 mg/100 g FW. Furthermore, the results illustrated that fruit harvested at full ripeness had the lowest phosphorus amount of 51.8 mg/100 g FW, followed by those harvested at semi-ripeness with a content of 90.7 mg/100 g FW. Fruit harvested at the semi-ripe stage, with a value of 207.6 mg/100 g FW, exhibited a higher phosphorus content compared to other maturation stages. It has been well-established that phosphorus accumulation in fruit follows a dynamic, stage-specific pattern influenced by the plant’s metabolic requirements for energy and cellular growth (31). For this investigation, the difference between the highest phosphorus content (207.6) in semi-ripe and the lowest (51.8) is 155.8 mg/100 g FW, which is significant. Variation in phosphorus content in fruit harvested at different stages could be attributed to the fact that intense metabolic demands are required to build and provide energy needed for fruit expansion. During this active development phase, the fruit functions as a major absorber organ, meaning energy production and nutrient allocation are at an absolute peak during the green unripe stage, increase in the semi-ripe stage and then drop when sugar accumulation begins.

In terms of human health and nutrition, phosphorus is essential because it contributes to the development of robust bones and teeth, facilitates cellular energy production and aids in the creation of genetic components (66). In this study, the difference between the highest phosphorus content (207.6) in green unripe fruit and the ARDI (369.8) is 162.2 mg. This suggests that pulp from green unripe fruit may potentially contribute about 56% of phosphorus required by humans daily. In addition, findings suggest that consumption of marula fruit at the green unripe stage could potentially assist in mitigating conditions such as loss of appetite, constant body pains, weak muscles and neurological issues, all of which are symptoms associated with a low phosphorus diet in humans (61, 67).

Regarding sodium content, results ranged from 10.1 to 22.4 mg/100 g FW. Additionally, results indicated that fruit harvested during the fully ripe stage had the lowest content of 10.1 mg/100 g FW, followed by those harvested at the semi-ripe stage with 15.2 mg/100 g FW. Compared with other maturity stages, the green unripe stage exhibited a superior sodium content of 22.4 mg/100 g FW. It has been established that sodium accumulation is a highly regulated process governed by absorption, xylem transport and phloem mobility at various maturity stages (68). In this investigation, the variation between the highest sodium content (22.4) in green unripe fruit and the lowest (10.1) is 12.3 mg/100 g FW. The fluctuation in sodium levels during different maturity stages of fruit is due to sodium’s limited mobility in the phloem, whereas the accumulation of sugars and water absorption rise, leading to an increase in fruit size and sugar concentration. The interplay of the increased water absorption and sugar accumulation during fruit maturation creates a dilution effect, thereby reducing salt uptake through these channels. These findings align with those of (69), who reported alterations in the metabolomic profile of tomato fruit exposed to different levels of salt stress.

Concerning human health and nutrition, sodium is an essential mineral and a key electrolyte that regulates fluid balance, maintains blood pressure and supports nerve and muscle function (70). For this investigation, the variation between the highest sodium content (22.4) and the ARDI (1500) is 1477.6 mg. This implies that pulp derived from green unripe marula fruit could potentially contribute about 1.5% of sodium required by humans daily. Although values obtained from this investigation are lower than the ARDI, consumption of pulps derived from green unripe fruit could potentially help curb conditions such as constant fatigue, muscle cramps and digestive complications, all of which are symptoms associated with low sodium intake in the human diet (71).

#### 3.3.2 Multivariate analysis of macro-minerals changes during marula fruit maturation (PCA and heatmap for ca, Mg, P, Na and K)

The PCA and heatmap analyses of microminerals provided further insight into mineral redistribution during fruit maturation (Figures 10, 11). The PCA biplot showed distinct clustering of samples according to maturity stage, with calcium, phosphorus and sodium being more closely associated with green unripe fruit, whereas magnesium was more closely aligned with fully ripe fruit. The first two principal components (PC1 and PC2) explained 78.9 and 11.2% of the total variance, respectively (Figure 10). The corresponding PCA loading plot (Supplementary Figure S3) showed that magnesium and calcium were the principal contributors to the positive direction of PC1, whereas phosphorus and potassium contributed to the opposite direction. Calcium and sodium also exhibited relatively strong negative loadings on PC2, indicating that these minerals contributed substantially to variation along the second principal component. The association of calcium and phosphorus with the green unripe stage is consistent with their essential roles in cell wall development, membrane integrity and rapidly dividing tissues during the early stages of fruit growth (68). Similar decreases in calcium and phosphorus during fruit ripening have been reported in several fruits and are generally attributed to dilution effects, nutrient redistribution and reduced accumulation as fruit expansion progresses (56). In contrast, the closer association of magnesium with the fully ripe stage suggests its continued involvement in metabolic processes associated with fruit maturation. Magnesium serves as a cofactor for numerous enzymes involved in carbohydrate metabolism and energy production, which become increasingly important during ripening as sugars accumulate and respiratory metabolism changes (63). The heatmap (Figure 11), further supported these observations, showing that calcium, phosphorus and sodium clustered predominantly with the green unripe stage, whereas magnesium clustered with the fully ripe stage. Collectively, these findings demonstrate coordinated changes in mineral composition during fruit maturation, reflecting the dynamic physiological and biochemical processes that accompany fruit development and ripening.

**Figure 10:**
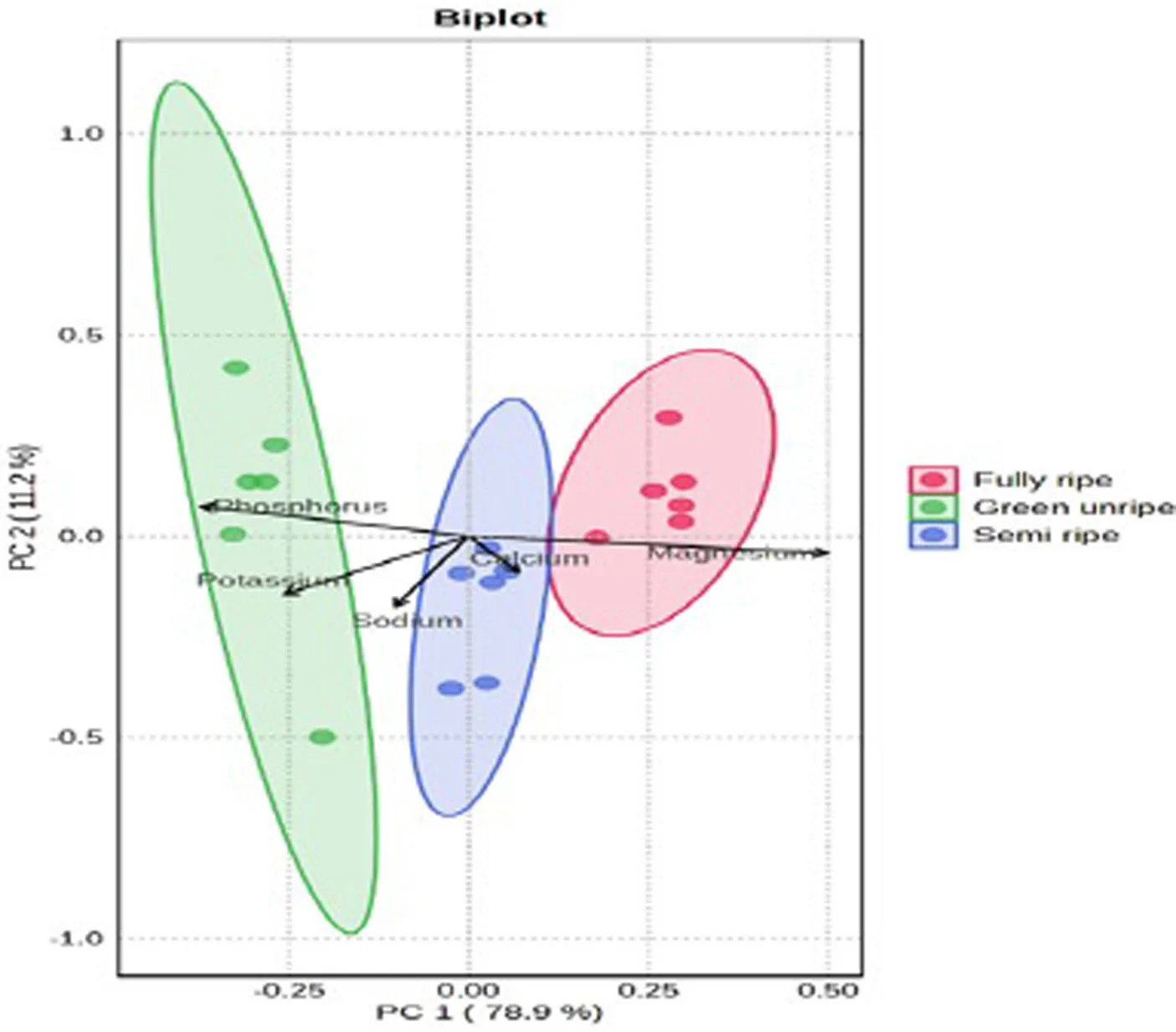
PCA biplot showing variation and correlation among macrominerals (P, K, Na, Ca, Mg) across different maturity stages of the marula fruit.

**Figure 11:**
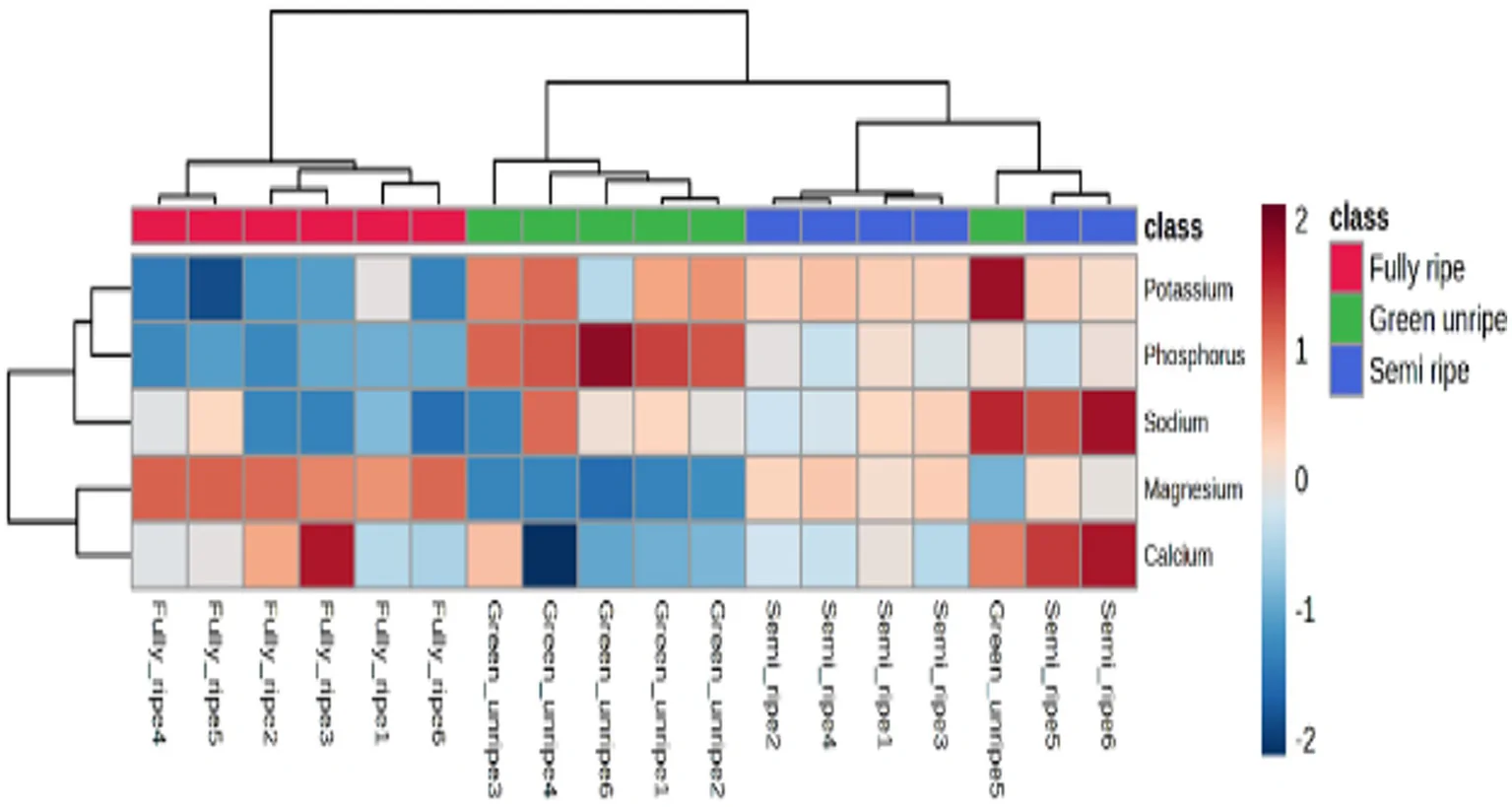
Heatmap representing relative concentrations and clustering patterns of the macrominerals across the different maturity stages of the marula fruit.

#### 3.3.3 Microminerals composition

Micronutrient (copper, iron and zinc) constituents of marula fruit harvested at varying maturity stages (green unripe, semi-ripe and fully ripe) are shown in Table 4. Study results demonstrated a significant (*p* ≤ 0.001) variation in micromineral (copper, iron and zinc) content across the different maturity stages. For copper, results ranged from 0.8 to 2.7 mg/100 g FW. In addition, study results showed that green unripe fruit had the lowest copper content of 0.8 mg/100 g FW, followed by those harvested at the semi-ripe stage with 1.3 mg/100 g FW. Compared with the other stages, fruit harvested at the fully ripe stage exhibited a super copper content of 2.7 mg/100 g FW. It has been well established that copper accumulation in fruit predominantly occurs during early cell division and expansion, as it is essential for enzyme activation and cell wall building, but its accumulation rate varies as developmental stages progress (53). For this investigation, the difference between the highest copper content (2.7) in fully ripe fruit and the lowest (0.8) in green unripe fruit was 1.9 mg/100 g FW, which was significant. The fluctuation in copper concentration in marula fruit at various developmental stages is ascribed to alterations in metabolic demand and heightened ethylene synthesis, as ethylene interacts with a specific protein receptor that requires copper as a cofactor to initiate ripening. These findings are in line with those of (72), who revealed copper as one of the main contributors to ethylene production in fruit during the maturity development stage.

**Table 4:** Effects of different maturity stages on the micromineral content of marula.

**Maturity stage**	**Copper**	**Iron**	**Zinc**
Green unripe	0.8 ± 0.3^c^	17.6 ± 1.2^a^	1.5 ± 0.5^a^
Semi-ripe	1.3 ± 0.1^b^	12.6 ± 1.9^b^	1.5 ± 0.5^a^
Fully ripe	2.7 ± 0.6^a^	8.6 ± 0.4^c^	0.9 ± 0.1^b^
Grand mean	1.572	12.9	1.617
LSD0.05	0.521	1.761	0.463
*P*-value	0.001	0.001	0.001

Values present the mean ± SE, *n* = 6; different letters within a column indicate significant differences (*p* ≤ 0.05).

In human health and nutrition, copper is an essential trace mineral crucial for the activity of several physiological enzymes (57). It is crucial for cellular energy production, iron metabolism, connective tissue synthesis, and the support of immunological and neurological activities (73). In this investigation, the variation between the highest copper content (2.7) and the recommended daily intake (1.1) is 1.6 mg. This suggests that pulps derived from fully ripe marula fruit could potentially provide copper levels that are double the recommended average daily consumption for people. Additionally, findings may suggest that consumption of marula fruit at the fully ripe stage could potentially help mitigate conditions such as chronic fatigue, frequent infections and poor bone health, which are all linked to insufficient copper intake in the human diet (74).

In terms of iron content, results ranged from 8.6 to 17.2 mg/100 g FW. Moreover, study results revealed that fruit harvested at the fully ripe stage had the lowest copper content of 8.6 mg/100 g FW, followed by those harvested at the semi-ripe stage at 12.6 mg/100 g FW. Compared with other maturity stages, fruit harvested at the green unripe stage had the highest copper content of 17.6 mg/100 g FW. It has been well established that iron accumulation in fruit is a highly regulated, stage-specific process linked to the plant’s nutritional requirements and structural development (75). For this investigation, the difference between the highest iron content (17.6) in green unripe fruit and the lowest (8.6) is 9 mg/100 g FW, which represents a reasonable margin. The fluctuation in iron concentration in fruit at various developmental phases is due to dilution as the fruit enlarges, driven by rapid water uptake and heightened sugar accumulation during maturation, which increases iron translocation and storage in the seeds. These findings align with those of (76), who reported a decrease in iron concentration in mulberry fruit across different developmental stages.

In human health and nutrition, iron is a crucial mineral because it supports critical biological functions, particularly oxygen transport, energy metabolism and neurological development (29). In this investigation, the variation between the highest iron content (17.6) in green unripe fruit and the ARDI (15.2) is 2.4 mg. This indicates that pulps derived from the green unripe marula fruit could potentially provide more than 100% of the daily iron required by humans. Additionally, values obtained from this study suggest that consumption of marula fruit pulp from green unripe fruit may potentially assist in curbing conditions such as constant fatigue, poor skin health, respiratory challenges and frequent headaches, which are conditions associated with low iron in the human diet (29, 77).

In terms of zinc content, results ranged from 0.9 to 1.5 mg/100 g FW. Additionally, compared with other stages, results evinced that fruit harvested at the fully ripe stage had the lowest zinc content of 0.9 mg/100 g FW. The highest zinc content was observed in fruit harvested at both the unripe and semi-ripe stages. It has been well established that during the fruit formation stage, the mineral zinc is often in greater demand to facilitate cell division in the fruit (78). Zinc is essential for protein synthesis, maintaining membrane integrity and producing hormones, such as auxins, which are crucial for structural growth (78). In this investigation, analysis revealed a significant difference of 0.6 mg/100 g FW between the highest zinc content (1.5) found in both green unripe and semi-ripe fruit and the lowest content (0.9) in fully ripe fruit. The observed change is due to the fruit expanding through water absorption and cellular enlargement, leading to a dilution effect from increased water content and sugar production, thereby significantly reducing zinc accumulation in fruit cells. These findings align with those of (57), who observed a significant reduction in zinc levels in tomato fruit across several developmental stages.

The mineral zinc is essential in human health and nutrition due to its ability to facilitate DNA synthesis, wound healing and the development of the embryo during pregnancy (79, 80). For this investigation, the variation between the highest zinc content (1.5) in both green unripe and semi-ripe fruit and the ARDI (9.1) is 7.6 mg. Values obtained from this study suggest that pulps derived from both green unripe and semi-ripe fruit could potentially contribute about 16.4% of zinc required by humans daily. Although values obtained from this study are lower in relation to the ARDI, consumption of marula fruit pulp at the green unripe and semi-ripe stages could potentially help curb conditions such as a weak immune system, hair loss, stunted growth and loss of taste, all of which are conditions associated with low zinc in the human diet (29, 81).

#### 3.3.4 Multivariate analysis of microminerals

Multivariate analysis of microminerals revealed distinct distribution patterns across the different maturity stages (Figures 12, 13). The PCA biplot showed clear separation of samples according to maturity stage, with iron being more closely associated with the green unripe fruit, whereas copper was more closely associated with the fully ripe fruit (Figure 12). The first two principal components (PC1 and PC2) explained 65.1 and 34.3% of the total variance, respectively. The corresponding PCA loading plot (Supplementary Figure S4) indicated that copper was the principal contributor to the positive direction of PC1, while iron contributed strongly to the opposite direction, demonstrating that these two elements were primarily responsible for the separation of maturity stages along the first principal component. Zinc exhibited a moderate positive loading on PC1 but the strongest negative loading on PC2, indicating that it contributed mainly to variation along the second principal component rather than to the primary separation of the maturity stages. The association of iron with the green unripe stage is consistent with its essential role in chlorophyll biosynthesis, electron transport and active cellular metabolism during early fruit development, when tissues exhibit high metabolic activity (82). Similar declines in iron concentration during ripening have been reported in other fruits and are generally attributed to nutrient redistribution and dilution associated with fruit expansion (83). In contrast, the closer association of copper with the fully ripe stage suggests its continued involvement in oxidative metabolism and copper-dependent enzymes that participate in respiration, antioxidant defense and cell wall modification during fruit ripening (82). Zinc showed comparatively less discrimination among maturity stages despite contributing to variation along PC2, suggesting that its concentration remained relatively stable throughout fruit development. The heatmap (Figure 13), further supported these observations by showing differential clustering of microminerals according to fruit maturity stage.

**Figure 12:**
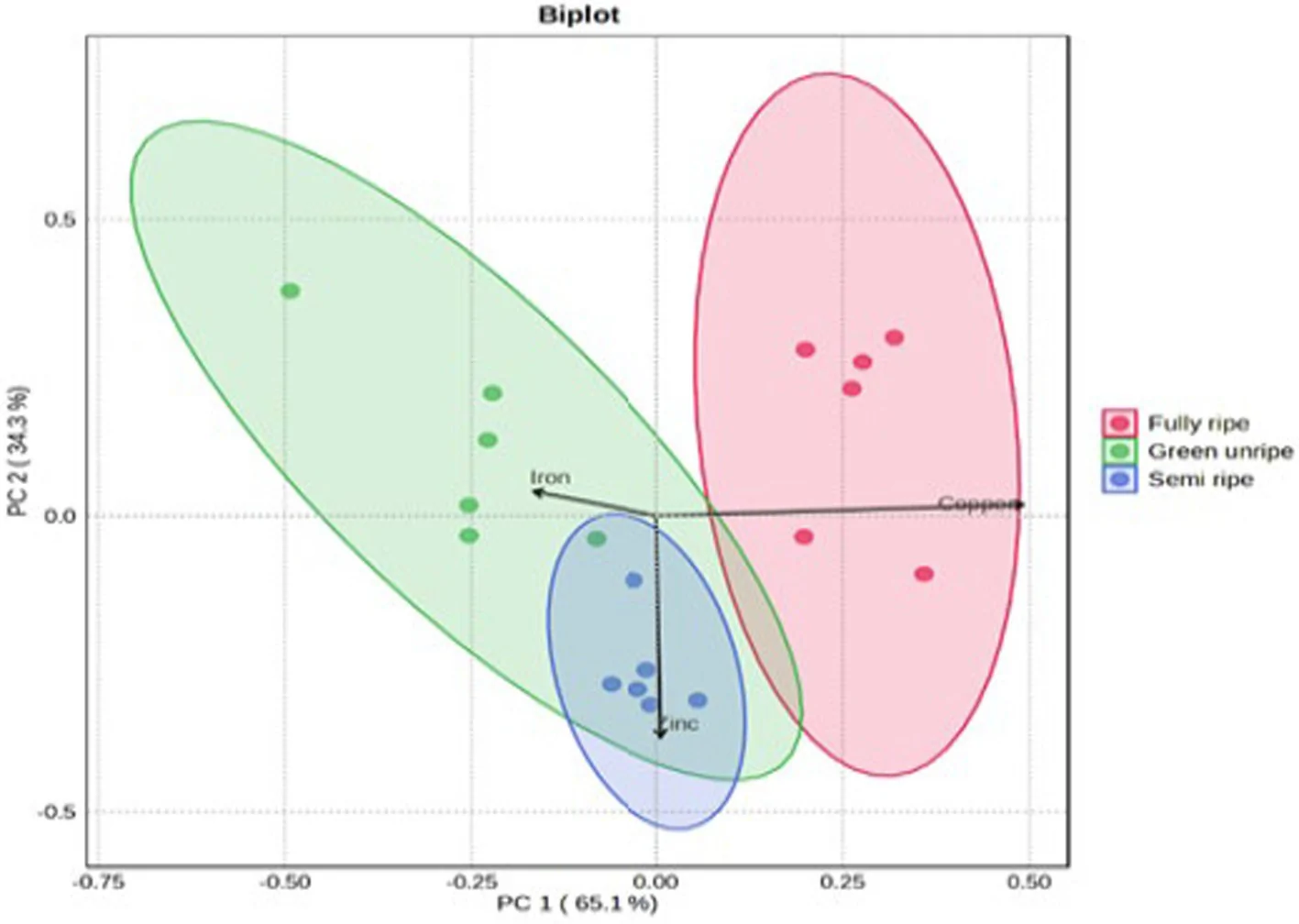
PCA biplot illustrating variation in micromineral composition (Fe, Zn, Cu) across the different maturity stages of the marula fruit.

**Figure 13:**
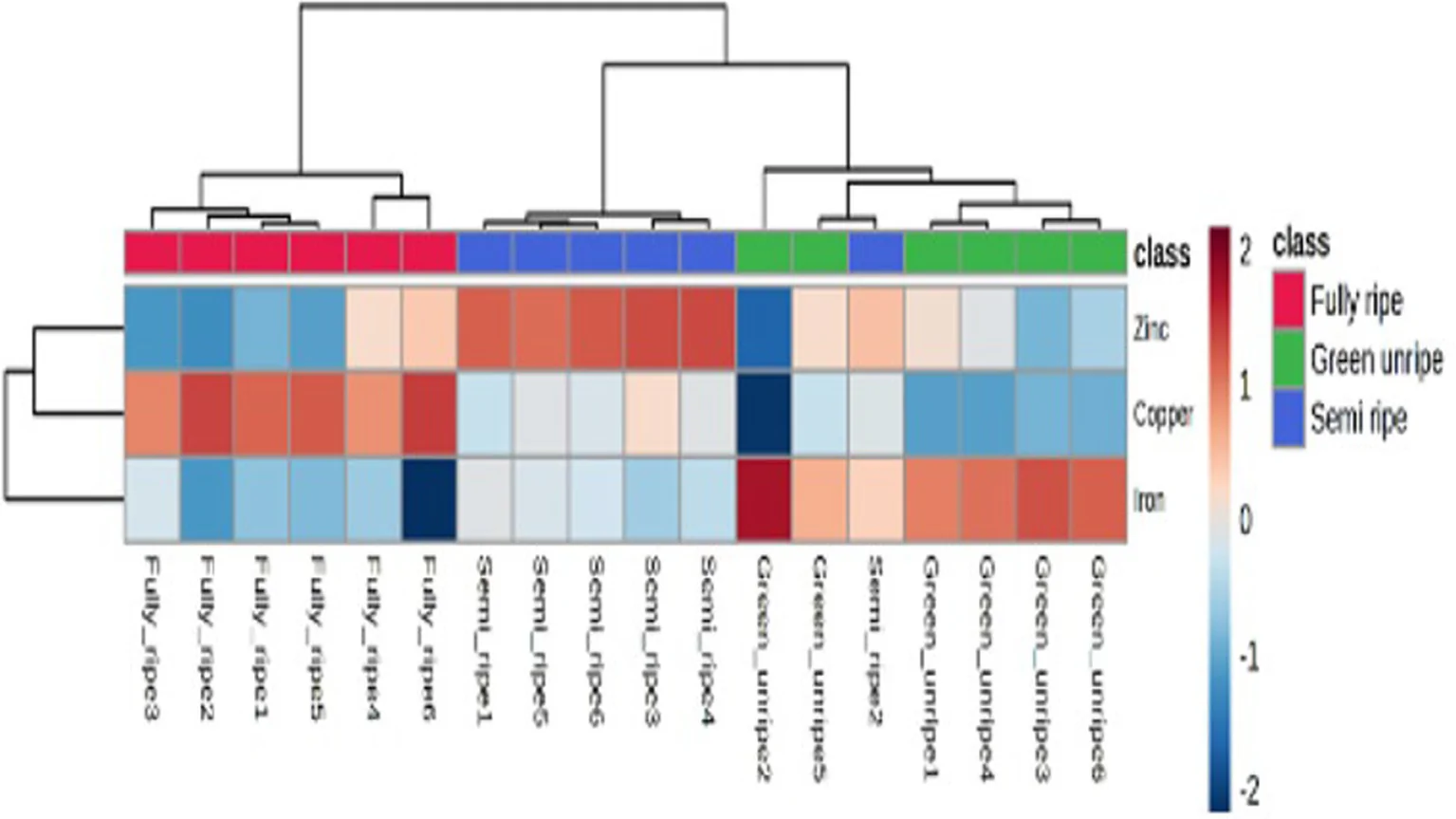
Heatmap showing clustering and relative abundance of microminerals across different maturity stages of marula fruit.

### 3.4 Integrated multivariate analysis of phytochemical and mineral composition of marula fruit

To provide a system-level understanding of compositional changes during marula fruit maturation, an integrated multivariate analysis combining physicochemical, phytochemical and mineral datasets was performed (Figure 14). The PCA biplot showed clear separation of the three maturity stages, with PC1 and PC2 explaining 77.6 and 11.9% of the total variance, respectively, indicating that most compositional variation during fruit maturation was captured by the first two principal components. The integrated PCA further demonstrated coordinated relationships among physicochemical, phytochemical and mineral variables. The loading vectors indicated that total flavonoids and phosphorus were positively associated with the green unripe stage, whereas β-carotene, copper, sugar-to-acid ratio and total soluble solids were associated with the fully ripe stage (Figure 14). Vitamin C and zinc were more closely associated with the semi-ripe stage, suggesting that this intermediate stage possesses a distinct compositional profile during fruit development. The association of green unripe fruit with acidity, phenolic compounds and selected minerals reflects the physiological requirements of actively developing fruit, where structural integrity, antioxidant defense and growth processes predominate (82, 83). The association of fully ripe fruit with sugars, carotenoids and copper is consistent with the biochemical changes accompanying ripening, including sugar accumulation, pigment development and enhanced nutritional quality. The positioning of the semi-ripe fruit between the green and fully ripe stages highlights the progressive nature of fruit maturation, during which compositional changes occur in a coordinated manner rather than independently. In summary, the integrated PCA demonstrates that physicochemical, phytochemical and mineral traits change simultaneously during ripening, providing a comprehensive representation of the biochemical progression underlying marula fruit maturation.

**Figure 14:**
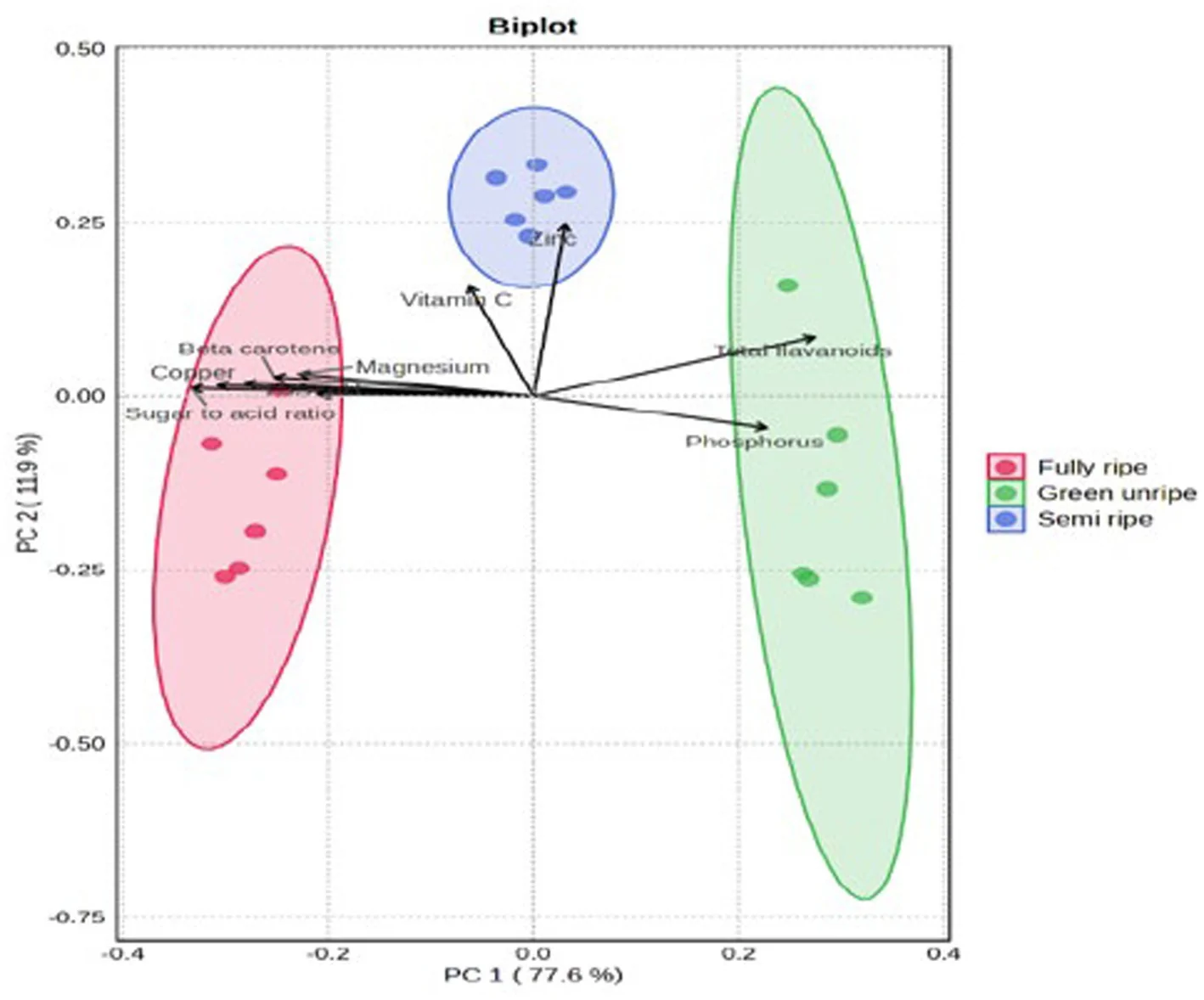
Principal component analysis biplot showing integrated variation in physicochemical, phytochemical and mineral composition of marula fruit across different maturity stages (green unripe, semi-ripe and fully ripe).

## 4 Potential practical application

- Improved shelf life and storage

Harvesting indigenous fruit at various stages of ripeness would aid in identifying optimal firmness, improving resilience to handling and storage, and therefore, significantly extending shelf life. Fruit remaining on the tree beyond optimal ripeness may quickly softened, sometimes with physical defects, rendering them vulnerable to bruising, pest infestations and fungal diseases. Therefore, determining the ideal maturity for harvesting would be crucial to enhancing postharvest practices and facilitating the attainment of desirable, edible qualities, thereby accelerating their commercial potential.

- Enhanced nutritional attributes

Harvesting at optimal maturity would ensure the highest level of physicochemical constituents, including total soluble solids, sugars, vitamin C, total flavonoids and phenols, all of which are essential for daily human consumption. Conversely, overripe fruit may exhibit increased sweetness as starches are converted to sugars, organic acids diminish and fermentation commences, eventually leading to rapid deterioration of the physicochemical properties. Therefore, determining the optimal harvesting stage for a specific use would potentially enable food producers to achieve superior-quality fruit suitable for that purpose, thereby minimizing losses.

- Processing efficiency

Fruit harvested at peak maturity offers the ideal balance of sugars, pectin and acids required for making jams, preserves or fruit leathers. In contrast, the high sugar-to-acid ratio often results in overly sticky or caramelized final products. Furthermore, damaged tissues are more prone to microbial contamination and crusting during sun-drying. Therefore, the timely harvesting of marula fruit at optimal maturity would potentially be essential for producing high-quality value-added goods, since it determines the produce’s sugar content, acidity, texture and flavor. Moreover, it would ensure that the raw material exhibits consistent reaction during processing, thereby leading to enhanced yields, extended shelf life and improved customer appeal.

- Marketability and consumer preference

Consumers are frequently categorized into various groups, with some favoring tougher, less-ripe fruit, while others prefer soft, fully ripe fruit. Consistent grading standards ensure that consumers are well informed of what to anticipate. Determining the marula fruit’s maturity stage could enhance consumer confidence, enabling retail businesses to embrace the fruit as a viable commercial product. This, in turn, would potentially allow harvesters and prospective producers to command premium prices, while reducing the incidence of unpalatable or damaged fruit, thereby mitigating food waste and increasing consumer willingness to pay.

- Improved food safety and public health

Fruit harvested at optimal physiological maturity retains excellent firmness and undamaged cell walls, hence reducing the risk of fungal and bacterial pathogens infiltrating the flesh. As a result, establishing the optimal harvesting maturity stage of marula fruit is crucial for rural and semi-urban food safety and public health, as it ensures maximum tissue integrity, thereby minimizing potential damage from microbial invasion and physical deterioration.

- Economic emancipation and formalization

Incorporating indigenous crops, such as marula fruit, into legislative frameworks such as the (Marketing of Agricultural Products Act) in South Africa and other parts of the world will potentially facilitate the formalization of rural value chains, potentially infusing some money benefits into the economy, while empowering local communities by enabling them to produce and sell goods to sustain their livelihoods.

## 5 Conclusion

The findings of this study indicate that the maturity stage of marula fruit significantly influences its physicochemical, phytochemical, and mineral composition. The compositional differences observed among the maturity stages suggest that green unripe fruit is characterized by higher concentrations of antioxidant-related phytochemicals, whereas fully ripe fruit contains higher β-carotene concentrations. These findings provide valuable baseline information for selecting harvest maturity according to the intended nutritional and processing application. Therefore, according to the finding’s marula fruit represents a promising source of essential nutrients and bioactive compounds. Furthermore, the study demonstrates the influence of harvest maturity on the nutritional composition of marula fruit and provides a scientific basis for future nutritional, postharvest, and utilization studies. While the findings provide valuable baseline information on maturity-associated compositional changes in marula fruit, further validation across multiple production environments and seasons would strengthen the broader applicability of these results. Although these findings highlight the nutritional potential of marula fruit, further studies evaluating nutrient bioavailability and postharvest performance are required before broader nutritional recommendations can be made.

## 6 Limitations

This study provides valuable discoveries into the maturity-related changes in the physicochemical, phytochemical and mineral composition of marula fruit. However, several limitations should be acknowledged. Fruit samples were collected from a single geographical location during one harvesting season, and therefore the findings may not fully capture the influence of environmental conditions, seasonal variability or genetic diversity on fruit composition. In addition, although the nutritional composition of the fruit was comprehensively characterized, the bioavailability and biological activity of the identified nutrients and phytochemicals were not evaluated. Furthermore, postharvest storage and processing conditions were not investigated, and these factors may influence the stability and retention of nutritional compounds. Future studies should therefore validate these findings across multiple locations and seasons, while incorporating bioavailability studies and postharvest storage and processing evaluations to better support harvest recommendations and value-added utilization of marula fruit. Carrying out such investigation may expedite the realization of the United Nations Sustainable Development Goals (SDGs), specifically SDG 1 – No Poverty, SDG 2 – Zero Hunger, SDG 3 – Good Health and Well-being, and SDG 4 – Responsible Consumption and Production.

## Data Availability

The raw data supporting the conclusions of this article will be made available by the authors, without undue reservation.
